# m^6^A-mediated lnc-OXAR promotes oxaliplatin resistance by enhancing Ku70 stability in non-alcoholic steatohepatitis-related hepatocellular carcinoma

**DOI:** 10.1186/s13046-024-03134-4

**Published:** 2024-07-25

**Authors:** Zhu Lin, Zhenkun Huang, Jiliang Qiu, Yunxing Shi, Dinglan Zuo, Zhiyu Qiu, Wei He, Yi Niu, Yunfei Yuan, Binkui Li

**Affiliations:** 1 State Key Laboratory of Oncology in South China and Collaborative Innovation Center for Cancer Medicine, Sun Yat-Sen University Cancer Center, Guangzhou, China; 2 Department of Liver Surgery, Sun Yat-Sen University Cancer Center, Guangzhou, China

**Keywords:** HCC, Long non-coding RNA, NASH, m^6^A, Oxaliplatin resistance

## Abstract

**Background:**

The escalating prevalence of metabolic diseases has led to a rapid increase in non-alcoholic steatohepatitis (NASH)-related hepatocellular carcinoma (NASH-HCC). While oxaliplatin (OXA)-based hepatic arterial infusion chemotherapy (HAIC) has shown promise in advanced-stage HCC patients, its efficacy in NASH-HCC remains uncertain. This study aims to assess the effectiveness of OXA-based HAIC and elucidate the mechanisms underlying OXA resistance in NASH-HCC.

**Methods:**

The key lncRNAs were screened through RNA-seq analysis of NASH/non-NASH and OXA-sensitive/OXA-resistant (OXA-S/R) HCC tissues. The biological functions of the lnc-OXAR (OXA resistance–related lncRNA in NASH-HCC) in NASH-HCC were verified through a series of in vitro and in vivo experiments. The molecular mechanism of lnc-OXAR was elucidated by fluorescence in situ hybridization, immunoprecipitation-mass spectrometry (FISH), Immunoprecipitation-Mass Spectrometry (IP-MS), RNA pulldown, RNA immunoprecipitation (RIP), methylated RNA immunoprecipitation sequencing (MeRIP-Seq) and a dual-luciferase reporter assay.

**Results:**

NASH-HCC exhibited reduced responsiveness to OXA-based HAIC compared to non-NASH HCC. We identified and validated a novel transcript namedlnc-OXAR, which played a crucial role in conferring OXA resistance to NASH-HCC. Inhibition of lnc-OXAR suppressed HCC cell growth and restored OXA sensitivity both in NASH-HCC mouse models and in vitro. Mechanistically, lnc-OXAR recruited Ku70 and cystatin A (CSTA), preventing Ku70 degradation and facilitating DNA double-strand break (DSB) repair, thereby promoting OXA resistance in NASH-HCC. Additionally, WTAP-mediated m^6^A modification enhanced the stability of lnc-OXAR in an IGF2BP2-dependent manner. Notably, silencing lnc-OXAR significantly enhanced the response to OXA in patient-derived xenograft (PDX) models derived from NASH-HCC.

**Conclusions:**

The reduced responsiveness of NASH-HCC to OXA treatment can be attributed to the upregulation of lnc-OXAR. Our findings provide a rationale for stratifying HCC patients undergoing OXA-based HAIC based on etiology. Lnc-OXAR holds promise as a novel target for overcoming OXA resistance in NASH-HCC and improving prognosis.

**Supplementary Information:**

The online version contains supplementary material available at 10.1186/s13046-024-03134-4.

## Background

The global epidemic of metabolic syndrome markedly increases the prevalence of non-alcoholic fatty liver disease (NAFLD), affecting one in four individuals in the general population [1]. Hepatic steatosis often exists in a clinically benign state but frequently progresses to non-alcoholic steatohepatitis (NASH), which increases the risk for end-stage liver diseases, such as liver cancer [2]. Currently, the estimated annual incidence of HCC ranges from 0.5 to 2.6% among patients with NASH cirrhosis and is expected to rise further [3]. NASH is the fastest growing cause of HCC according to a recent estimate [4]. Unlike other types of HCC, NASH-HCC shows distinctive genius morbid and development mechanism, which might have varying degree of response to different therapies [5]. For example, preliminary data from preclinical studies suggest that immunotherapy efficacy may be reduced in NASH-related HCC compared to viral HCC [6].

Hepatic arterial infusion chemotherapy (HAIC) allows direct delivery of chemotherapeutic agents into tumors feeding hepatic arteries at increased local concentration. There is growing evidence demonstrating the efficacy of oxaliplatin (OXA)-based HAIC in patients with advanced-stage HCC [7, 8]. OXA-based HAIC has shown promising clinical outcome with higher objective response rate and prolonged progression-free survival (PFS) [9, 10]. However, more than half of treated patients are likely to not respond and ultimately succumb to their disease, leading to limited clinical application [10]. Therefore, biomarkers or clinical characteristics which could effectively predict the efficacy of OXA treatment are crucial for patient selection. Interestingly, here we found that HCC with a background of NASH is less responsive to OXA-based HAIC. Based on the clinical findings, it was further verified the resistance of NASH-HCC to OXA-based HAIC in vivo. Therefore, we were particularly interested in elucidating the mechanism of OXA resistance in NASH-HCC patients to provide novel strategies for overcoming it in clinical practice.

Long noncoding RNAs (lncRNAs) is a major class of noncoding RNAs with a length of more than 200 nt. In the past decade, the focus in cancer genomics has shifted towards the noncoding part of the genome, especially on the lncRNAs, which have proved to be involved in the regulation of several malignant processes [11]. Furthermore, lncRNAs has recently emerged as a significant contributor in the NASH-HCC progression [12]. It was reported that lncRNA SNHG6 increased progression from NAFLD to HCC by modulating cholesterol-induced mTORC1 activation [13]. Regarding the regulation of OXA resistance in HCC, lncRNA LINC01134 regulated HCC-OXA resistance through SP1-induced p62 transcription as reported by a previous study [14]. Nevertheless, a functional role of lncRNAs promoting OXA resistance in NASH-HCC has not been well described.

In the present study, combining analysis of clinical data and experimental data from mouse NASH-HCC models, we found that NASH-HCC patients are less responsive to OXA-based HAIC than non-NASH-HCC patients. We identified that the lnc-OXAR (OXA resistance–related lncRNA in NASH-HCC) was upregulated in NASH-HCC patients as well as OXA resistant patients. Lnc-OXAR has a pivotal role in OXA resistance of NASH-HCC by enhancing Ku70 stability to protect cancer cells from double-strand DNA breaks (DSBs). Upstream, m^6^A methylated modification could mediate lnc-OXAR upregulation via WTAP and insulin like growth factor 2 mRNA binding protein 2 (IGF2BP2). Collectively, our findings revealed lnc-OXAR as a key regulator in NASH-HCC OXA resistance and provided attractive strategies to overcome OXA resistance in the treatment of NASH-HCC.

## Methods

### Mice

Mice were purchased from Guangdong Medical Laboratory Animal Center.

### Nude mice subcutaneous xenograft model

Four-week-old BALB/c male nude mice (*n* = 5 per group) with the average body weight of 18 to 22 g were randomly divided into groups. Xenografts were initiated by subcutaneous injection of 2 × 10^6^ PLC/PRF/5 or Huh-7 or 8 × 10^6^ HepG2 cells (on the right sides) into the back of nude mice. About thirty days later, the mice were sacrificed and the tumor nodules were harvested and photographed.

### Orthotopic xenograft mouse model

For orthotopic xenograft mouse model, mice were fed ad libitum either a normal chow diet plus drinking water or a modified western diet (Envigo-TD.120528) plus sugar water (23.1 g/L fructose and 18.9 g/L glucose) for 2 weeks prior to implantation. After anaesthetizing and exposing the liver, Huh-7 cells (2 × 10^6^) in 50 μl PBS solution were orthotopically injected into left lobe of the liver using a 50 μL Hamilton microliter syringe, and the incision was closed using surgery suture threads with needle. GS or OXA (10 mg/kg) was intraperitoneally administered into the mice twice a week for at 14 days post implantation. At the experimental endpoint, mice tumors were monitored by the in vivo imaging system (IVIS) system after luciferin injection for 15 min. Tumor tissues were harvested for further analysis.

### Patient-Derived tumor Xenograft (PDX) mouse model

For PDX tumor, specimens were obtained from patients with histologically confirmed HCC. All tumors were collected in accordance with the institutional review board-approved protocols of the Sun Yat-sen Cancer Center (SYSUCC). PDX tumors were generated by transplanting small tumor fragments isolated directly from surgical specimens subcutaneously into nude mice. Each tumor was allowed to grow until 1 cm, after which it was harvested. 10% of this tumor was re-implanted in a nude mouse, and the tumor was thus propagated for 3 ~ 4 generations until it was used for this experiment. 5 ~ 6 nude mice were randomly grouped and implanted HCC-PDX. GS or OXA (10 mg/kg) were intraperitoneally administered into the mice twice a week when the tumors reached about 100 mm^3^ in size. We began to perform intratumoral injections of scrambled or in vivo-optimized lnc-OXAR inhibitor (5 nmol per injection, RiboBio, Guangzhou, China) every 3 days for 20 days. When the study finished, the mice were anesthetized, and the tumor volume and weight were measured. All tissues from the cell-based xenografts or PDXs underwent further pathological analysis.

### Cell model construction

After the growth density of HepG2 cells reached 70%–80% according to the manufacturer’s instructions, approximately 1 × 10^5^ cells were inoculated in 6-well plates and incubated for 24 h. The NAFLD model was established by a complete culture medium containing 250 μM sodium oleate and 122.5 μM sodium palmitate for 48 h. A complete culture medium supplemented with solvent was used as the control group (Control-HepG2 group). The lipid accumulation level was detected by oil red O staining.

### Human tissue specimens and HCC tissue microarray

All patients received curative surgical resection from January 2015 to December 2020 and were confirmed as NASH-HCC or non-NASH-HCC by postoperative pathological diagnosis. Human HCC tissues and matched adjacent non-tumor liver tissues were obtained from patients who received curative surgery at the SYSUCC. The related clinicopathological features of the enrolled patients are presented in Table S2. All samples were obtained with the informed consent of the patients. This study complied with the standards of the 1975 Declaration of Helsinki and the experiments were approved by the Ethics Committee of SYSUCC.

### Organoid culture

Organoids were cultured from HCC patients with a tumor size of 500 mm^3^. Briefly, dissected tumors were finely minced and transferred to a 50 mL centrifuge tube, including a digestion mix consisting of Ad-DMEM/F-12 medium (Gibco, USA) and 1 mg/mL collagenase IV (Sigma, USA), and incubated for 40 min at 37 ◦C. Isolated organoids were mixed with 5 μL of Matrigel (Costar, USA) and seeded in 96-well plates (Costar, USA). The culture medium contains Ad-DMEM/ F-12 with B27 supplement (1 ×), nicotinamide (10 mM), N-acetyl-Lcysteine (1.25 mM), EGF (5 ng/ml), A83- 01 (500 nM), SB202190 (10 μM), Y-27632 (10 μM), Noggin (100 ng/mL), R-Spondin 3 (250 ng/ml), FGF2 (5 ng/ml), FGF 10 (10 ng/ml), penicillin/streptomycin (1 ×) and Glutamine (1 ×). Supplemented culture medium (100 μL) was added per well, and organoids were maintained in a 37 ℃ humidified atmosphere under 5% CO2.

### Organoid viability

Organoids were seeded into 96-well plates at 300–500 organoids in 5 μL of Matrigel per well in a total volume of 100 μL of the medium. Serially diluted compounds in 100 μL of medium were added to the cells 24 h later. After 4 days of incubation, Cell-Titer Glo reagents (Promega, USA) were added, and luminescence was measured. After 4 days of incubation, the medium was carefully aspirated and 100 μL of live/dead reagents (UElandy, China) was added followed by 30 min of incubation at room temperature. A fluorescence microscope was used to capture images of calcein AM (494/517 nm) to represent the live cells, of PI (535/617 nm) to identify the dead cells. The above assays were performed in triplicates.

### Bioinformatics analysis

Data from TCGA (https://portal.gdc.cancer.gov/) or RNA-Seq were analyzed by R (V3.3, http://www.bioconductor.org) with the edge R package. Fold-change (FC) of gene expression was calculated with threshold criteria of FC ≥ 2 and *P* value < 0.05. GO enrichment analysis was performed to investigate the processes of the candidate genes or metabolites, by applying online tools of the DAVID (https://david.ncifcrf.gov/).

Additional Materials and Methods can be found in the Supplementary Materials.

## The information of antibody and prime are provided in Supplementary Materials

### Statistical analysis

The experiments were repeated at least three times independently, and the measured data were represented as the mean ± SD. Binary variables were compared by using the Chi-squared test, and ordinal categorical variables were compared by the Kruskal–Wallis test. Survival curves were constructed using the Kaplan–Meier method and analyzed by the log-rank test. Significant prognostic factors found by univariate analysis were entered into a multivariate analysis using the Cox proportional hazards model. The correlations between lnc-OXAR and Ku70 were assessed by Spearman correlation analysis. All analyses were two-sided, and differences with *P* values less than 0.05 were considered significant. Statistical analyses were performed using the GraphPad Prism 9.0 software (GraphPad, Inc., La Jolla, CA, USA).

## Results

### NASH-HCC was less responsive to OXA-based HAIC

**Fig. 1:**
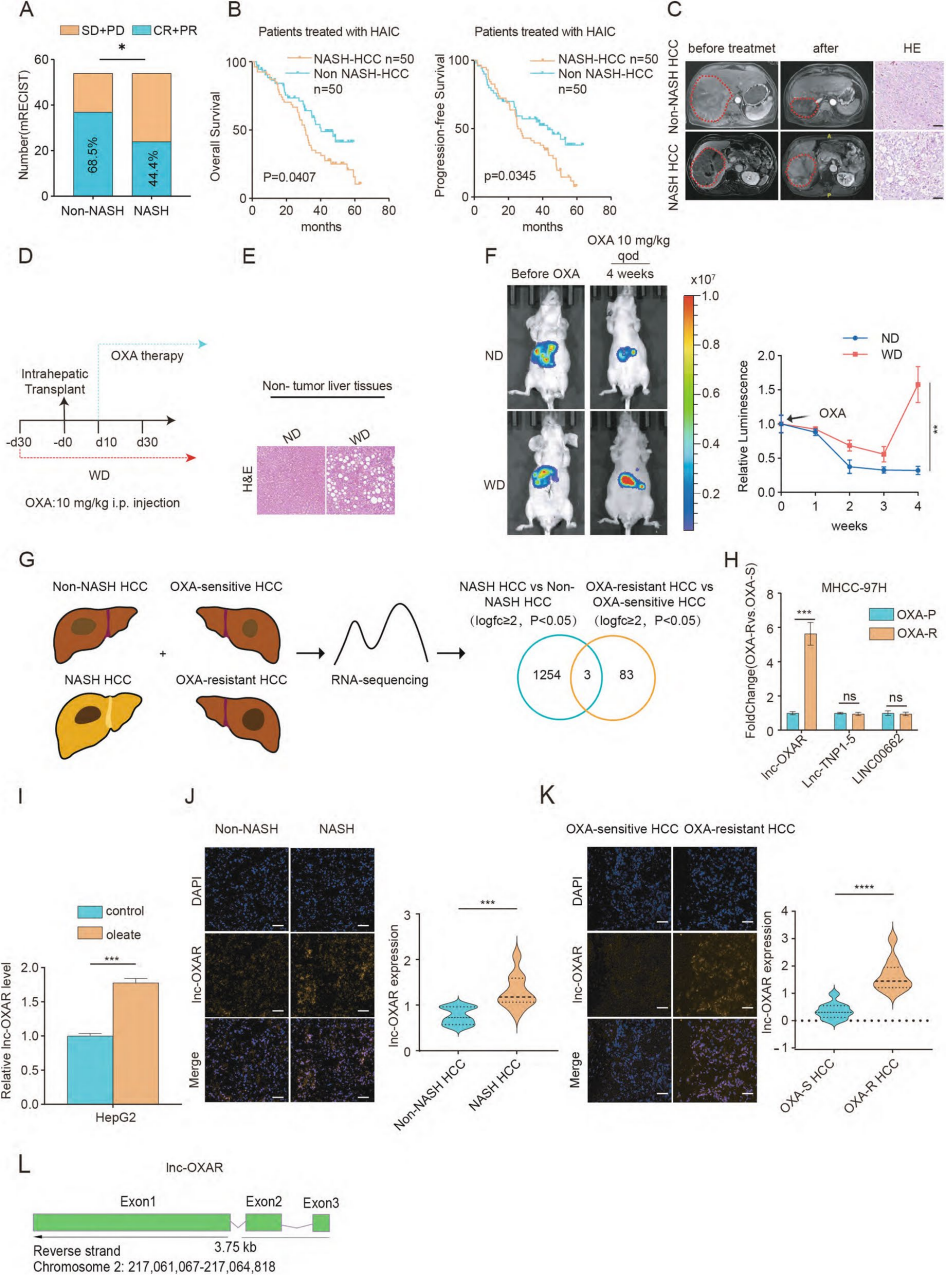
Identification of lnc-OXAR as an OXA resistance–related lncRNA in NASH-HCC. **A** Patients who received OXA-based HAIC were separately divided into NASH-HCC and Non-NASH HCC based on surgical pathologic findings. Kaplan–Meier survival curves and log-rank tests were used to compare OS and PFS between the two groups. **B** The responses of the SYSUCC HCC cohort to OXA-based HAIC were determined by mRECIST criteria. **C** MRI images of NASH-HCC and Non-NASH HCC before and after OXA-based HAIC treatment. Representative HE staining of NASH and non-NASH patients. Scale bar = 50 μm. **D** Timeline schematic of the NASH- HCC model. i.p injection, intraperitoneal injection. GS, glucose solution. ND, Normal Diet. WD, Western Diet. **E** Representative images of HE-stained non-tumor livers from orthotopic NASH-HCC mice fed a normal diet and western diet. Scale bar = 50 μm. **F** Representative images of tumor burden post-intrahepatic injection (Huh-7) for orthotopic HCC mice fed a control diet or western diet and treated with OXA using in vivo bioluminescent imaging. And measurement of tumor burden weekly. **G** Venn diagram showing candidate lncRNAs involved in OXA-resistance based on RNA-seq analysis of OXA resistant HCC and NASH-HCC patient samples. **H** Quantitative real- time PCR (qPCR) analysis of the candidate lncRNAs between OXA- P and OXA-R cells. **I** lnc-OXAR expression was detected in HepG2 cells treated with vehicle or oleate. **J** lnc-OXAR expression was detected in Non-NASH HCC and NASH HCC tissues by FISH. Histogram reflects the relative lnc-OXAR fluorescence intensity evaluated from 10 different fields of vision. Scale bar = 50 μm. **K** lnc-OXAR expression was detected in OXA-sensitive (OXA-S) and OXA-resistant (OXA-R) HCC tissues by FISH. Histogram reflects the relative lnc-OXAR fluorescence intensity evaluated from 10 different fields of vision. Scale bar = 50 μm. **L** lnc-OXAR is located on Chromosome 2: 217,061,067–217,064,818 and has 3 exons

To study the efficacy of OXA-based HAIC in NASH-HCC, patients who had received OXA-based HAIC were stratified into NASH-HCC and non-NASH-HCC groups based on histological findings demonstrating hepatocellular ballooning degeneration and hepatic lobular inflammation with hepatic steatosis [15]. Responses were assessed using modified Response Evaluation Criteria in Solid Tumors (mRECIST), and the objective response rate was lower in the NASH-HCC group than in the non-NASH-HCC group (ORR, 44.4% vs 68.5%, *P* < 0.05) (Fig. 1A). In the NASH-HCC group, patients had poorer overall survival and progression-free survival than those in the non-NASH-HCC group (Fig. 1B). The decrease in tumor volume was more prominent in non-NASH HCC after OXA-based HAIC, as shown in Fig. 1C. The above results implied that NASH-HCC patients were less responsive to OXA-based HAIC.

Furtherly, we established an orthotopic mouse model using the Huh-7 HCC cell line and fed the mice with western diet or normal diet (Fig. 1D). HE staining of liver tissues showed western diet induced hepatocyte steatosis (Fig. 1E). Weight gain and larger tumors were observed in western diet group compared with normal diet controls (Supplementary Fig. 1A). Moreover, mice fed with western diet bore larger tumors compared with controls after OXA treatment (Fig. 1F). Western diet group showed increased Ki67-positive cells and a clear reduction in γH2AX (a DNA damage marker) foci of tumors treated with OXA (Supplementary Fig. 1B), suggesting that NASH-HCC was less sensitive to OXA in vivo.

### Identification of lnc-OXAR as an OXA resistance-related lncRNA in NASH-HCC

To investigate critical lncRNAs that are potentially associated with OXA resistance in NASH-HCC, we performed RNA-sequencing (RNA-seq) analysis in NASH-HCC vs non-NASH-HCC tissues (log2FC ≥ 1, *P* ≤ 0.01) and OXA-sensitive (OXA-S) vs OXA-resistant (OXA-R) HCC tissues (log2FC ≥ 1, *P* ≤ 0.01) (Fig. 1G). Eighty-six lncRNAs significantly upregulated in OXA-R tissues (Supplementary Fig. 1C, D). And 1,257 transcripts were upregulated in the NASH-HCC tissues (Supplementary Fig. 1E). After overlapping two sets of screening data, three lncRNAs were identified. To further screen differentially expressed lncRNAs, we constructed OXA-resistant cells (OXA-R) from parental MHCC-97H cells (Supplementary Fig. 1F). Half maximal inhibitory concentration (IC50) and apoptosis assay showed that the OXA-R cell line was successfully established (Supplementary Fig. 1G-H). The up-regulated candidate lncRNAs were further validated in OXA-R HCC cell line (Fig. 1H). Lnc-OXAR was the only up-regulated lncRNA in OXA-cells among the three candidate lncRNAs.

Subsequently, to investigate the expression of lnc-OXAR in NASH-HCC cells, the NASH model was established by oleate induction in HepG2 cells and found a significant upregulation in oleate-treated HepG2 cells (Fig. 1I). Fluorescence in situ hybridization (FISH) analysis further revealed the upregulation of lnc-OXAR in NASH-HCC as well as OXA-R patients (Fig. 1J, K). Bioinformatics analysis revealed that lnc-OXAR is located on Chromosome 2: 217,061,067–217,064,818 and has 3 exons. (Fig. 1L). Taken together, these results suggest lnc-OXAR might serve as the critical lncRNA in NASH-HCC OXA resistance.

### Lnc-OXAR promoted OXA resistance in NASH-HCC.

**Fig. 2:**
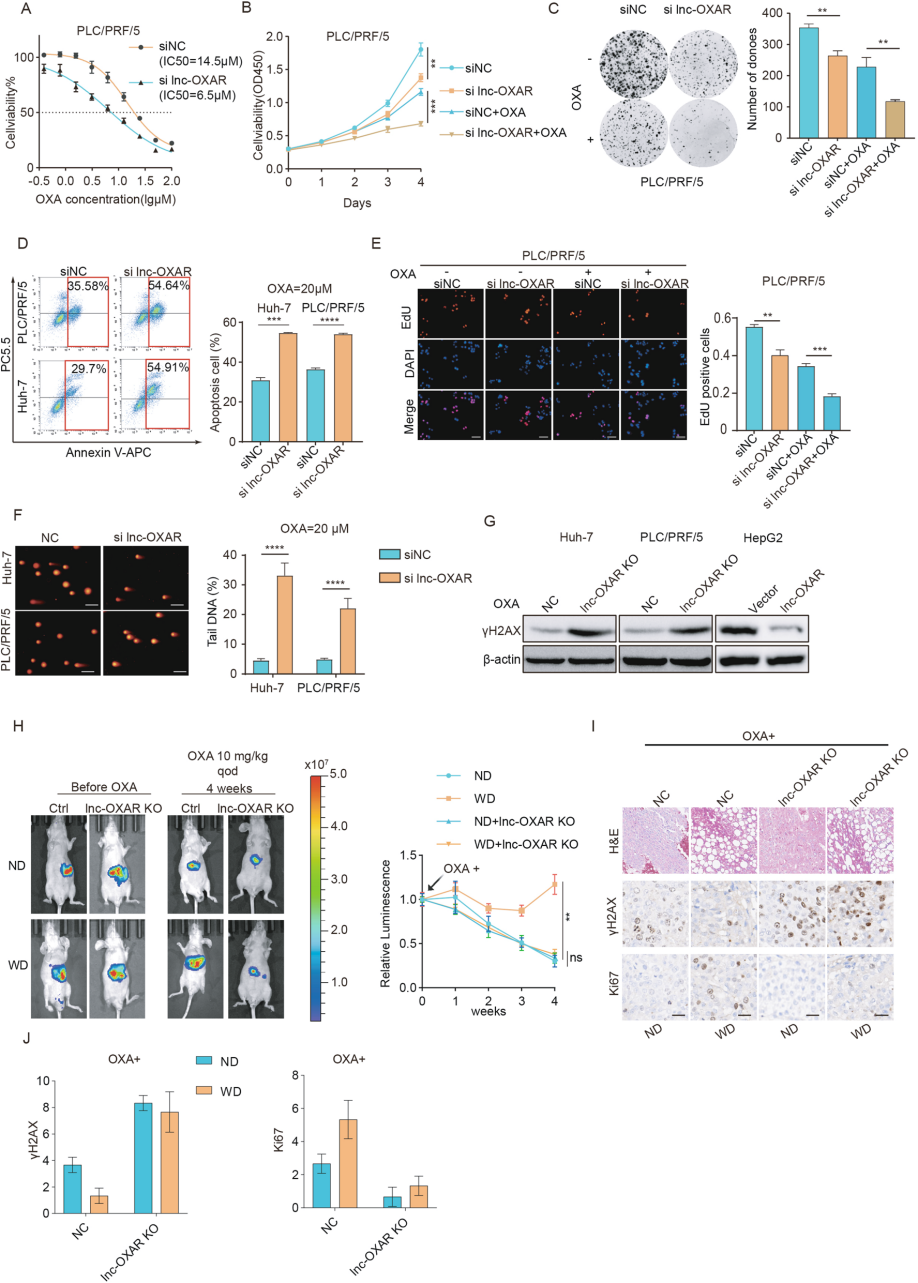
lnc-OXAR promoted OXA resistance in NASH HCC. **A** Cell viability curves of HCC cells transfected with siNC and si lnc-OXAR. Cells without OXA are presented as 100% and used as an internal control. **B** Cell proliferation was assessed by CCK8 assays (OD 450 nm) in HCC cells transfected with siNC and si lnc-OXAR. **C** Colony formation assays and statistical analysis of HCC cells transduced with siNC or si lnc-OXAR. **D** The effect of lnc-OXAR knockdown on apoptosis in PLC/PRF/5 and Huh7 cells treated with oxaliplatin (20 μM, 48 h). **E** EdU detection in PLC/PRF/5 cells transfected with siNC and si lnc-OXAR with or without OXA treatment (10 μM, 48 h). Scale Bar = 50 μm. **F** Representative images of comet assay of HCC cells transfected with siNC and si lnc- OXAR with OXA treatment (20 μM, 48 h) and quantitative analysis. Scale bar = 20 μm. **G** WB analysis detecting γH2AX inHCC cells transduced with lnc-OXAR KO or NC or control vector or vector expressing lnc-OXAR. **H** Representative images of tumor burden post-intrahepatic injection (Huh-7 NC or lnc-OXAR KO) for orthotopic HCC mice fed a control diet or western diet and treated with OXA using in vivo bioluminescent imaging. And measurement of tumor burden weekly. **I** HE staining and IHC staining of Ki67 and γH2AX in the tumors. Representative images of xenografts from each group are shown. Scale bar = 50 μm. **J** Quantification of IHC analysis

To investigate the functional roles of lnc-OXAR in OXA resistance, we screened several HCC cell lines and found that lnc-OXAR expression was detected in all 8 cell lines (Supplementary Fig. 2A). Subsequent evaluation of OXA sensitivity in these HCC cell lines revealed a positive correlation between lnc-OXAR expression and OXA resistance (Supplementary Fig. 2B, C). Since lnc-OXAR was confirmed to exhibit the highest expression in PLC/PRF/5 and Huh-7 cells and the lowest expression in HepG2 cells, we silenced lnc-OXAR in PLC/PRF/5 and Huh-7 cells and overexpressed in HepG2 cells (Supplementary Fig. 2D, E). For lnc-OXAR knockdown, we employed a smart silencer composed of three siRNAs and three antisense oligonucleotides (ASOs). Lnc-OXAR knockdown not only decelerated cell growth but also increased the sensitivity of HCC cells to OXA, which were demonstrated by Cell Counting Kit-8 (CCK8) assay (Fig. 2A, B and Supplementary Fig. 2F-G), colony-formation assay (Fig. 2C and Supplementary Fig. 2H), apoptosis assay (Fig. 2D) and 5-ethynyl-2′-deoxyuridine (EdU) incorporation by fluorescence microscopy (Fig. 2E, Supplementary Fig. 2I). OXA can induce DSBs by forming platinum–DNA adducts [16]. To assess OXA-induced DSBs, comet assay and γH2AX expression assay were conducted. Results showed an increased DSB level after lnc-OXAR knockdown (Fig. 2F, G). Overexpression of lnc-OXAR produced the opposite results (Supplementary Fig. 2 J-O).

To further investigate the role of lnc-OXAR in the resistance of NASH-HCC to OXA treatment, we generated lnc-OXAR KO cells using the CRISPR/Cas9 genome-editing system (Supplementary Fig. 2P). Then, we established an orthotopic mouse model using the lnc-OXAR KO cells and fed them with western diet or normal diet. The sensitivity to OXA was restored by lnc-OXAR knockout in western diet-fed mice(Fig. 2H). Western diet increased Ki67-positive cells and reduced γH2AX foci in tumors treated with OXA, and these effects could be attenuated by lnc-OXAR deletion (Fig. 2I, J).

We also performed a subcutaneous tumor formation assay to study the function of lnc-OXAR in vivo. Antagonizing lnc-OXAR significantly decreases cancer cell proliferation, and enhanced OXA sensitivity in vivo which were consistent with the in vitro results (Supplementary Fig. 3A, B). Conversely, tumors with lnc-OXAR overexpressed were significantly larger than control and became less sensitive to OXA treatment (Supplementary Fig. 3C). Collectively, these results suggest that lnc-OXAR played an important role in promoting OXA resistance in NASH-HCC.

### Lnc-OXAR interacted with and maintained Ku70 stability by recruiting CSTA

**Fig. 3:**
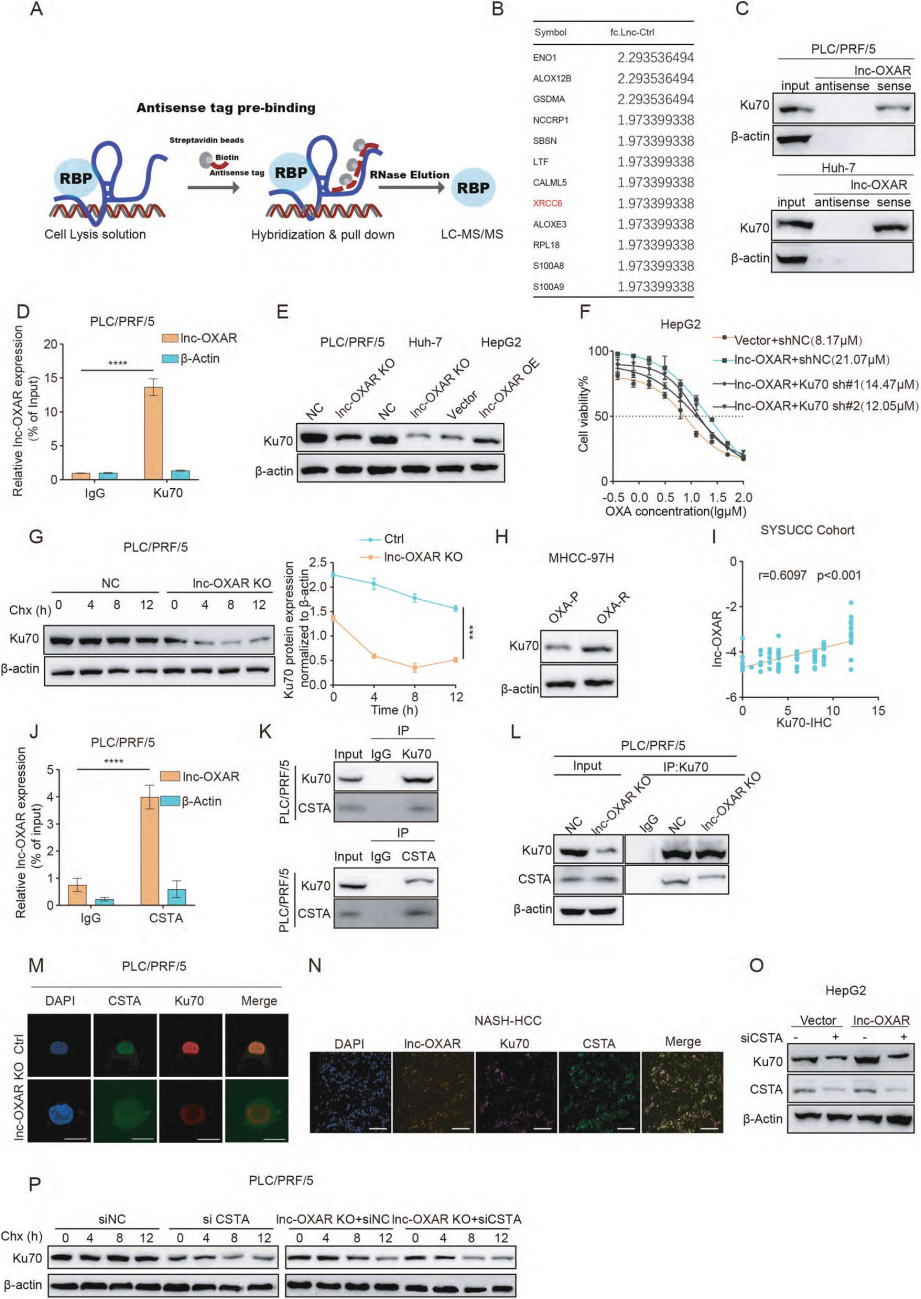
lnc-OXAR maintained Ku70 stability by recruiting CSTA. **A** Overview of the ChIRP-MS method used to identify lnc-OXAR-interacting proteins. **B** The top 15 peptide fragments based on foldchange of proteins pulled down by lnc-OXAR in PLC/PRF/5 cells. **C** RNA pulldown of lnc-OXAR sense or antisense with Ku70. **D** Ku70 RIP-qPCR analysis of lnc-OXAR level in PLC/PRF/5 cells. **E** The expression levels of Ku70 in HCC cells with or without lnc-OXAR KO (or lnc-OXAR OE). **F** The effect of Ku70 KD on the IC50 of lnc-OXAR-overexpressing in HepG2 cells. **G** WB showing Ku70 protein in PLC/PRF/5 cells with or without lnc-OXAR knockout treated with CHX for the indicated time (left). The quantification of Ku70 degradation rate by gray scale analysis (right). **H** WB analysis of Ku70 level in MHCC-97H OXA-P and OXA-R cells. **I** Pearson correlation analysis of Ku70 protein level and lnc-OXAR RNA level in SYSUCC cohort, determined using qPCR and IHC. **J** CSTA RIP-qPCR analysis of lnc-OXAR level in PLC/PRF/5 cells. **K** WB results verified that Ku70 associated CSTA. **L** WB of immunoprecipitated Ku70 and CSTA to determine the effect of lnc-OXAR KO on PLC/PRF/5 cells. **M** Immunofluorescence (IF) staining showed the co-localization of CSTA (green) and Ku70 (red) in HCC cells. Scale bar = 10 μm. **N** IF/FISH reveals distributions of lnc-OXAR, Ku70 and CSTA in the NASH-HCC tissue. Scale bar = 50 µm. **O** WB analysis of Ku70 to determine the effect of CSTA silencing in lnc-OXAR OE cells. **P** WB showing the effect of CSTA silencing on Ku70 protein in PLC/PRF/5 cells with or without lnc-OXAR knockout after treated with CHX for the indicated time, along with quantification of Ku70 degradation

To address how lnc-OXAR contributed to OXA resistance in NASH-HCC, we screened for lnc-OXAR-binding proteins using comprehensive identification of RNA-binding proteins by mass spectrometry (ChIRP-MS) (Fig. 3A) (Table S2). After initial screening based on unique peptides (≥ 2) and fold change (> 1.5), the top 15 proteins enriched in the ChIRP lysate were shown (Fig. 3B). The GO enrichment analysis was conducted on the ChIRP-MS results and revealed significant enrichment of double-strand break repair via nonhomologous end joining (NHEJ) including Ku70 (Supplementary Fig. 4A). In the NHEJ pathway, XRCC6 (Ku70) is the central core of NHEJ pathway. Oxaliplatin exerts an anticancer effect by forming platinum–DNA adducts and causing DNA damage and Ku70 is essential for DNA repair [17]. Therefore, we hypothesized that Ku70 might serve as a key downstream target of lnc-OXAR in OXA resistance in NASH-HCC.

To investigate whether Ku70 overexpression correlates with poor HCC prognosis, we detected the expression levels of Ku70 in HCC patients using immunohistochemistry (IHC). The level of Ku70 in the tumor was higher than in adjacent tissue in both TCGA and SYSUCC cohorts (Supplementary Fig. 4B, C). Additionally, overall survival (OS) and recurrence-free survival (RFS) were also shorter in those patients with high level of Ku70 (Supplementary Fig. 4D-F). A higher level of Ku70 was associated with higher clinical stage and alpha fetoprotein (AFP) level (Supplementary Fig. 4G, H). It revealed that Ku70 is mainly localized in the nucleus of cells (Supplementary Fig. 4I). To validate the specificity of Ku70-RNA binding, RNA-pulldown-wb was performed. We found that the sense strand of lnc-OXAR, but not the antisense control, specifically interacted with Ku70 (Fig. 3C). RIP-qPCR of Ku70 confirmed that Ku70 interacts with the lnc-OXAR (Fig. 3D and Supplementary Fig. 4 J, K). Next, we explored the correlation between Ku70 and lnc-OXAR expression. Lnc-OXAR was positively correlated with Ku70 protein level without affecting its mRNA level (Fig. 3E and Supplementary Fig. 4L). To investigate whether Ku70 is a downstream target of lnc-OXAR to promote the resistance of OXA, next we found knockdown of Ku70 could restore the sensitivity to OXA in lnc-OXAR OE cells (Fig. 3F). Moreover, lnc-OXAR knockout markedly decreased Ku70 protein expression with cycloheximide (CHX) treatment, suggesting a shorter half-life of Ku70 (Fig. 3G and Supplementary Fig. 4 M). While in lnc-OXAR OE cells, Ku70 levels remained more stable (Supplementary Fig. 4N), indicating a longer half-life of Ku70. We also found the protein levels of Ku70 in OXA-R cells were significantly higher than in OXA-P cells (Fig. 3H). Lnc-OXAR expression level strongly positively correlated with Ku70 protein level in patient tissues (Fig. 3I). Together these results demonstrate that lnc-OXAR interacts with Ku70 and is involved in its stability.

We further explored the mechanism by which the lnc-OXAR remained the stability of Ku70. In the results of ChIRP-MS, we found that lnc-OXAR interacts with CSTA (Table S2) and it has been reported to stabilize ITGB1 in HCC cells [18]. Interaction between lnc-OXAR and CSTA was confirmed by RIP-qPCR (Fig. 3J, and Supplementary Fig. 4O). Our co-IP assays found that Ku70 interacted with CSTA (Fig. 3K and Supplementary Fig. 4P). Lnc-OXAR KO cells showed reduced binding of CSTA and Ku70, indicating that the binding relied heavily on lnc-OXAR (Fig. 3L and Supplementary Fig. 4Q). The fluorescence profiles confirmed Ku70/CSTA subcellular colocalization (Fig. 3M and Supplementary Fig. 4R-S). We found the co-localization of lnc-OXAR, Ku70 and CSTA in HCC tissues through FISH-IF (Fig. 3N).

In order to investigate whether lnc-OXAR promoted the stabilization of Ku70 through CSTA, CSTA was knocked down in lnc-OXAR OE cells and the levels of Ku70 protein were analyzed. Interestingly, the Ku70 level was reduced in lnc-OXAR OE cells after CSTA knockdown, suggesting that lnc-OXAR could potentially stabilize Ku70 protein through CSTA (Fig. 3O). CSTA protein knockdown showed a shorter half-life of Ku70 protein in lnc-OXAR ctrl cells, which was not evident in lnc-OXAR KO cells (Fig. 3P). In both vector and lnc-OXAR OE cells, CSTA protein knockdown showed a significantly shorter half-life of Ku70 protein (Supplementary Fig. 4 T). Based on these results, lnc-OXAR interacts with Ku70 and maintains its stability by recruiting CSTA.

### The effect of lnc-OXAR on OXA resistance in NASH-HCC is dependent on Ku70.

**Fig. 4:**
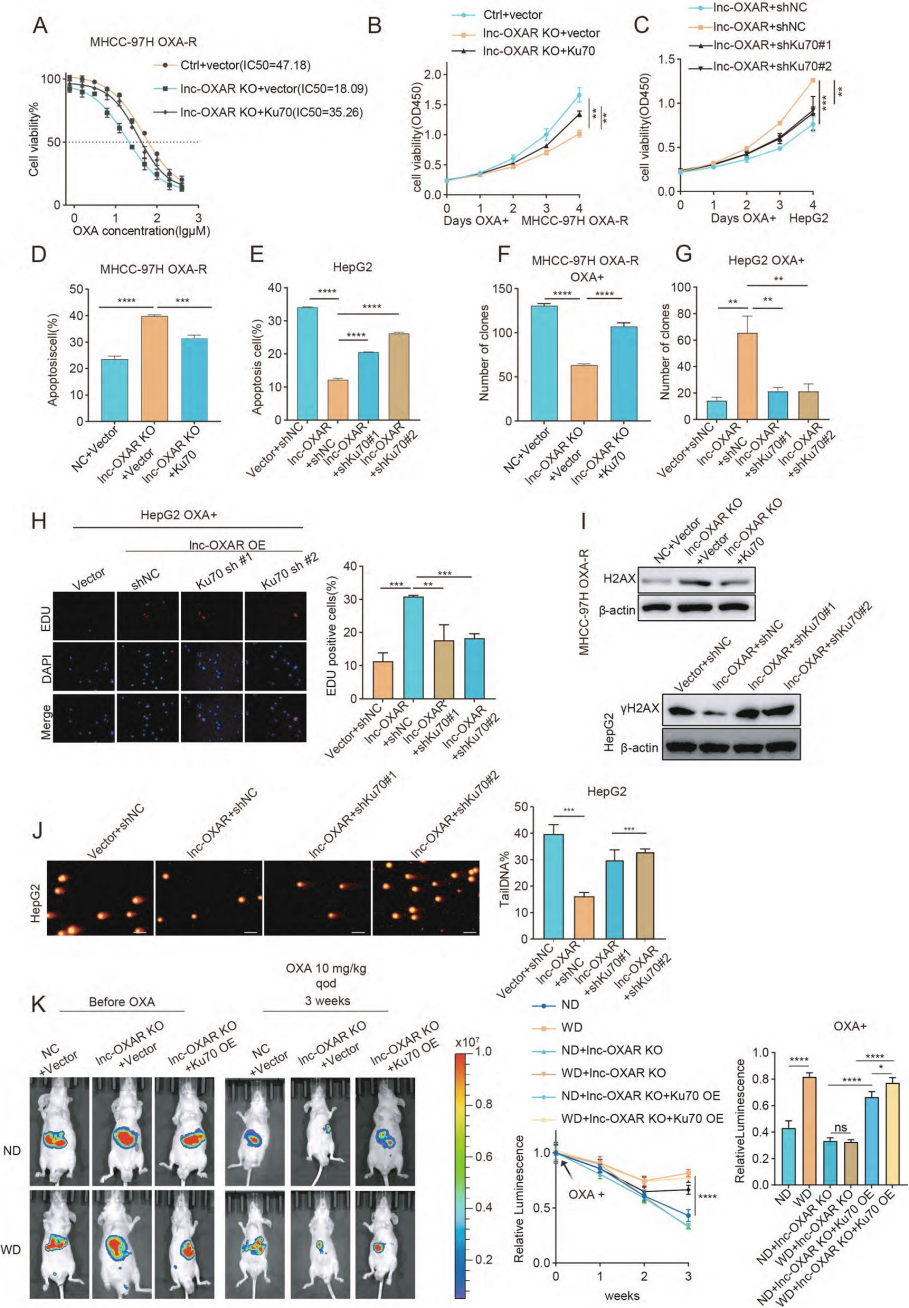
The effect of lnc-OXAR on OXA resistance in NASH-HCC is dependent on Ku70. **A** The effect of Ku70 OE on the IC50 of lnc-OXAR-KO in OXA-R cells. **B** The effect of Ku70 OE on the CCK8 assay of lnc-OXAR KO in OXA-R cells with OXA treatment (2 μM). **C** The effect of Ku70 KD on the CCK8 assay of lnc-OXAR-overexpressing in HepG2 cells with OXA treatment (2 μM). **D** The effect of Ku70 OE on the apoptosis of lnc-OXAR-KO in OXA-R cells with treatment (20 μM,48 h) using flow cytometry analysis of Annexin V staining. **E** The effect of Ku70 KD on the apoptosis of lnc-OXAR-overexpressing in HepG2 cells with treatment (20 μM,48 h) using flow cytometry analysis of Annexin V staining. **F** The effect of Ku70 OE on the colony-formation of lnc-OXAR KO in OXA-R cells with OXA treatment (5 μM). **G** The effect of Ku70 KD on the colony-formation of lnc-OXAR-overexpressing in HepG2 cells with OXA treatment (1 μM). **H** The effect of Ku70 KD on the EDU assay of lnc-OXAR-overexpressing in HepG2 cells with OXA treatment (2 μM). Scale bar = 50 μm. **I** The effect of Ku70 KD on the γH2AX level of lnc-OXAR-overexpressing in HepG2 cells with OXA treatment (20 μM). And the effect of Ku70 OE on the γH2AX level of lnc-OXAR KO in OXA-R cells with OXA treatment (20 μM). **J** The effect of Ku70 KD on the comet assay of lnc-OXAR-overexpressing in HepG2 cells with OXA treatment (20 μM,48 h). Scale Bar = 20 μm. **K** Representative images of tumor burden post-intrahepatic injection (Huh-7 NC or lnc-OXAR KO) for orthotopic HCC mice fed a control diet or western diet and treated with OXA using in vivo bioluminescent imaging. Measurement of tumor burden weekly. And average radiance of tumor burden at the end point

Since lnc-OXAR binds to Ku70 and positively regulates Ku70 protein expression, we further verified the hypothesis that Ku70 acts as the main downstream target of lnc-OXAR in regulating OXA-resistance in NASH-HCC. Compared to the control, Ku70 knockdown increased the sensitivity of PLC/PRF/5 cells to OXA (Supplementary Fig. 5A, B). Overexpression of Ku70 in lnc-OXAR KO OXA-R cells rescued the cell growth and restored resistance to OXA in OXA-R cells, and conversely, knockdown of Ku70 in lnc-OXAR OE cells reduced cell proliferation and restored its sensitivity to OXA (Fig. 4A-H, Supplementary Fig. 5C-J). Also, Ku70 knockdown in lnc-OXAR OE cells was associated with increased DNA damage as reflected in comet assay and γH2AX accumulation (Fig. 4I-J). Consistently, Ku70 OE robustly reduced the DNA damage in lnc-OXAR KO OXA-R cells, as evidenced by the decreased γH2AX level (Fig. 4I).

Next, we investigate the role of ku70 in lnc-OXAR-mediated OXA resistance in NASH-HCC in vivo. Overexpression of Ku70 restored the tumor growth activity and OXA resistance inhibited by lnc-OXAR KO (Fig. 4K, Supplementary Fig. 6A). Several molecular markers were examined by IHC (Supplementary Fig. 6B). Ku70 OE in lnc-OXAR KO cells showed dramatically increased Ki67-positive tumor cells compared to lnc-OXAR KO cells both with and without OXA treatment. Furthermore, Ku70 OE reduced the DNA damage effects induced by OXA in lnc-OXAR KO tumor. Taken together, these observations strongly suggest that the effect of lnc-OXAR on OXA resistance in NASH-HCC depends on Ku70.

### m^6^A modification mediated lnc-OXAR upregulation in NASH-HCC

**Fig. 5:**
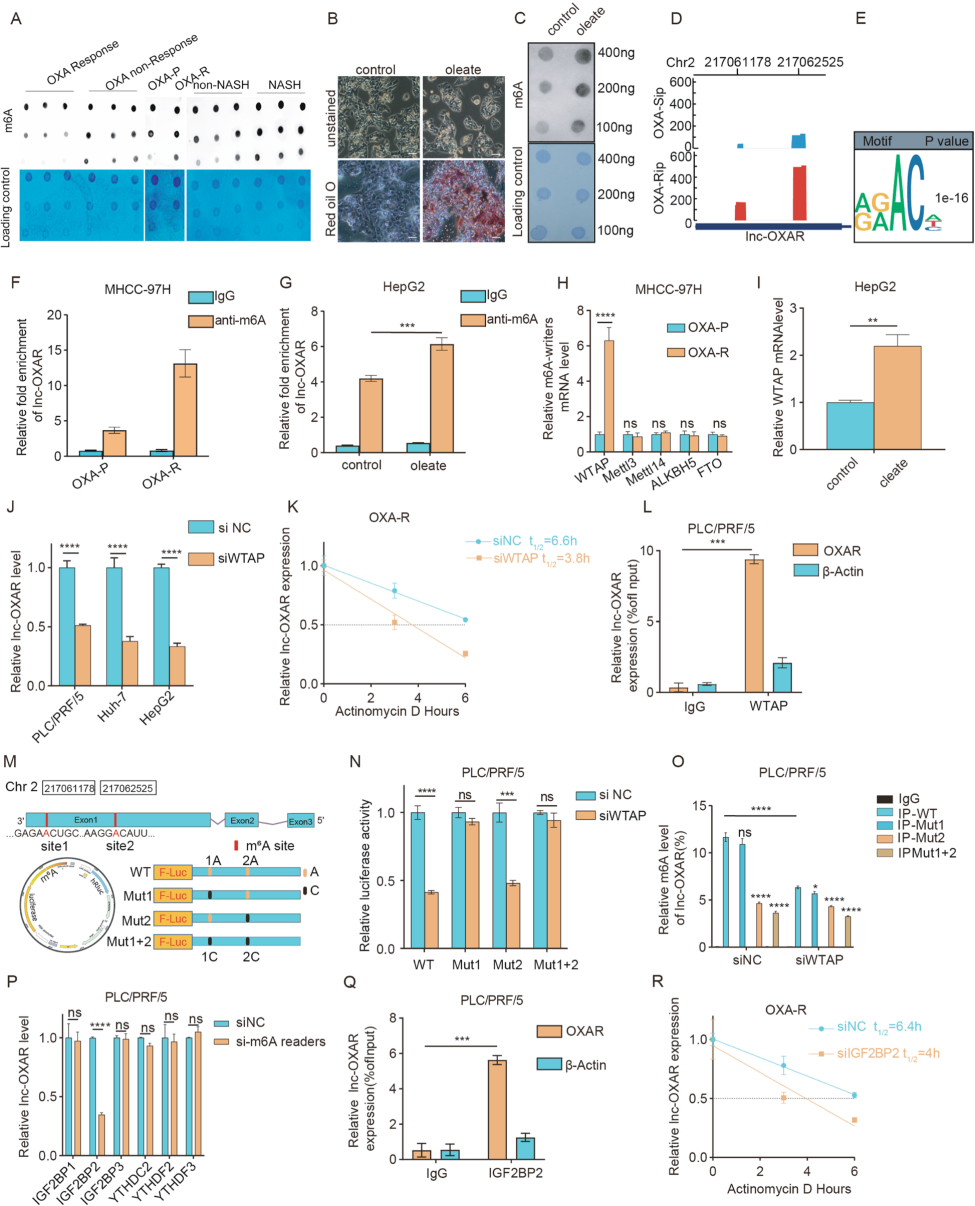
m^6^A modification mediated lnc-OXAR upregulation in NASH-HCC. **A** Global m^6^A levels were measured in parental and resistant (Non-NASH HCC and NASH HCC) tissues or cells using m^6^A dot blotting assays. **B** After oleate treatment, the cells were stained with Oil Red O dye; lipid droplets are visible in red. Scale bar = 20 μm. **C** Global m^6^A levels were measured in HCC cells with oleate treatment using m^6^A dot blotting assays. **D** Profile of m^6^A distribution on lnc-OXAR in OXA-P and OXA-R cells. **E** Representative motif of m^6^A modification of transcripts in OXA-R cells, two-sided Fisher’s exact test with adjustments for multiple testing. **F** m^6^A level of lnc-OXAR transcripts in MHCC97H OXA-P and OXA-R cells detected by Me-RIP-qPCR. **G** m^6^A level of lnc-OXAR transcripts in HepG2 cells treated with vehicle or oleate detected by Me-RIP-qPCR. **H** The expression of five m^6^A writers/erasers was detected by qPCR in OXA-P and OXA-R cells. **I** WTAP expression was detected in HepG2 cells treated with vehicle or oleate. **J** The expression of lnc-OXAR in HCC cells after WTAP silencing. **K** The degradation rate of lnc-OXAR after WTAP silencing in OXA-R cells with actinomycin D treatment. **L** WTAP RIP-qPCR analysis of lnc-OXAR level in PLC/PRF/5 cells. **M** Diagram showing the position of m^6^A motifs and corresponding mutant sites. **N** Two m^6^A modification sites were validated through step-by step mutation of luciferase reporter. **O** Dual-luciferase reporter assay of wild-type or site-mutant PLC/PRF/5 cells with or without WTAP silencing. **P** lnc-OXAR expression was detected after multiply silencing different m^6^A readers. **Q** IGF2BP2 RIP-qPCR analysis of lnc-OXAR level in PLC/PRF/5 cells. **R** The degradation rate of lnc-OXAR after IGF2BP2 silencing in OXA-R cells with actinomycin D treatment

To investigate the regulatory mechanism underlying lnc-OXAR upregulation in NASH-HCC, pre- and post-transcriptional regulatory mechanisms were further studied. There was no difference in lnc-OXAR expression after treatment with 5-azacytidine (5-AZA, DNA methylation inhibitor) or trichostatin A (TSA, histone acetylation inhibitor), indicating that neither of these mechanisms was responsible for lnc-OXAR upregulation (Supplementary Fig. 7A, B). The small molecule inhibitor 3-Deazadenosine (DAA), which inhibits m^6^A deposition by reducing levels of the methyl donor S-adenosylmethionine [19], substantially attenuated lnc-OXAR expression (Supplementary Fig. 7C). Thus, we focused on m^6^A modifications in lnc-OXAR upregulation in NASH-HCC. The global m^6^A modification level was detected by dot blot assays. Our results confirmed that m^6^A modification was indeed upregulated in OXA-R tissues, NASH-HCC tissues and OXA-R cells (Fig. 5A). To evaluate m^6^A modification level in steatosis, an oleate-treated HepG2 cell model was employed (Fig. 5B). Dot blot assay showed that m^6^A modification was also upregulated in HepG2 cells treated with oleate (Fig. 5C). Therefore, we found that high-fat environment could induce m^6^A modification upregulation in HCC.

Next, we examined whether the m^6^A level in the lnc-OXAR was elevated in OXA-R cells. Methylated RNA immunoprecipitation sequencing (Me-RIP-Seq) was performed. Peak distribution analysis showed that m^6^A sites were enriched in lnc-OXAR (Fig. 5D). The consensus motif of RRACH (R = purine, A = m^6^A, and H = A, C, or U) was identified in m^6^A-purified peaks (Fig. 5E). Me-RIP-qPCR showed that m^6^A level of lnc-OXAR was significantly higher in OXA-R and oleate treated HCC cells (Fig. 5F and G). NASH-HCC and OXA-resistant cells exhibited high amount of total m^6^A levels.

To understand the effects of m^6^A modification on lnc-OXAR expression, the expression of m^6^A writers and erasers was then evaluated in OXA-P HCC and OXA-R HCC cells. Among these m^6^A regulators, WTAP showed remarkably increased expression in OXA-R cells, which could be responsible for the upregulated m^6^A levels (Fig. 5H). Oleate-treated cells also displayed increased WTAP mRNA level (Fig. 5I). WTAP knockdown in HCC cells was then constructed (Supplementary Fig. 7D). Lnc-OXAR were significantly downregulated by WTAP knockdown (Fig. 5J). WTAP knockdown significantly decreased lnc-OXAR mRNA stability (Fig. 5K, and Supplementary Fig. 7E-G). The interactions between lnc-OXAR and WTAP were confirmed experimentally by RIP-qPCR (Fig. 5L). Moreover, the lnc-OXAR level was found to positively correlate with the protein level of WTAP in human NASH-HCC tissues (Supplementary Fig. 7H). So far, we had proved that WTAP-mediated m^6^A modification was the underlying cause of lnc-OXAR upregulation in NASH-HCC.

To further search for m^6^A sites in lnc-OXAR, we generated dual-luciferase reporter constructs containing firefly luciferase before the wild-type or mutant lnc-OXAR sequence with A at the m^6^A sites substituted with G (Fig. 5M). Dual-luciferase reporter showed that the site2 mutation decreased the m^6^A modification of lnc-OXAR with a significantly decreased expression level (Fig. 5N). These results were confirmed by Me-RIP-qPCR (Fig. 5O).

The fate of transcripts harboring m^6^A modification was determined by the recognized readers. To further identify the m^6^A reader that can recognize lnc-OXAR, different m^6^A modification readers were knocked down in HCC cells (Supplementary Fig. 7I). We found IGF2BP2 siRNA significantly downregulated level of lnc-OXAR (Fig. 5P). To validate the direct binding between IGF2BP2 protein and lnc-OXAR,The enrichment of lnc-OXAR was detected by RIP-qPCR to confirm the interaction between lnc-OXAR and IGF2BP2 (Fig. 5Q). The lnc-OXAR decay rate was significantly decreased after the knockdown of IGF2BP2 in HCC cells (Fig. 5R, and Supplementary Fig. 7 J-L). The lnc-OXAR level was found to positively correlate with the protein level of IGF2BP2 (Supplementary Fig. 7 M). Our data indicated that WTAP-mediated m^6^A modification of lnc-OXAR maintains its stability by preventing its degradation via the IGF2BP2-dependent pathway.

### Clinical significance of targeting lnc-OXAR in NASH-HCC

**Fig. 6:**
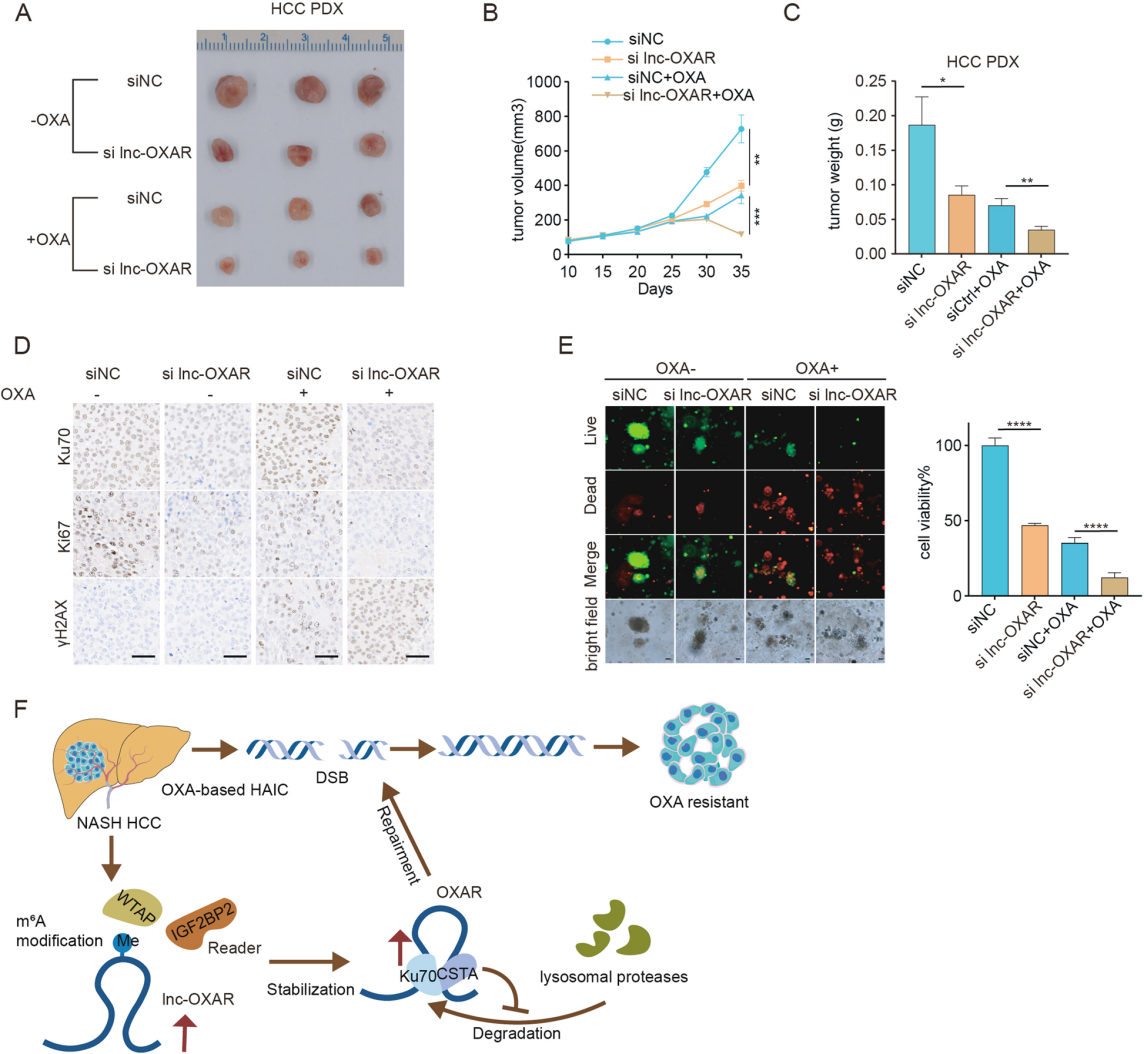
Clinical significance of targeting lnc-OXAR in NASH-HCC. **A** Images of extracted NASH-HCC PDX transduced with siNC or si lnc-OXAR with or without OXA treatment. **B** Tumor growth curve of PDX models. **C** Tumor weight of PDX models. **D** Representative IHC images for Ku70, Ki67 and γH2AX from PDX model. Scale bar = 50 μm. **E** NASH HCC patient-derived organoids were treated with vehicle or OXA. Four days later, representative images were taken under a fluorescence microscope or standard light microscope. Cell viability in organoids was measured with CellTiter-Glo. Scale bar = 100 μm. **F** Schematic diagram of the underlying mechanisms of lnc-OXAR in promoting OXA resistance in NASH HCC

To further confirm the significance of targeting lnc-OXAR in vivo, we constructed NASH-HCC patient-derived xenograft (PDX) models in nude mice and utilized antisense oligonucleotides (ASO) to knockdown nuclear-localized RNAs (Fig. 6A). As a result of lnc-OXAR knockdown, the trend of umor growth was significantly inhibited with OXA treatment (Fig. 6B-C). Accordingly, as shown in the IHC analyses, lnc-OXAR knockdown significantly decreased Ku70 protein level and increased the level of OXA-induced DSBs (indicated by γH2AX). Besides, lnc-OXAR knockdown significantly reduced cell proliferation (indicated by Ki67), especially with the OXA treatment (Fig. 6D). We also found that knockdown of lnc-OXAR expression with ASO significantly reduced organoid formation and lnc-OXAR knockdown sensitized cells to OXA (Fig. 6E). Thus, lnc-OXAR might serve as an attractive therapeutic target for NASH-HCC patients to overcome OXA resistance.

## Discussion

Currently, NASH-HCC is treated according to the same guidelines for other etiologies [20]. HCC on the background of NASH presents additional considerations because of the crosstalk between inflammatory and metabolic environment manifest in the disease such as insulin resistance, steatosis, oxidative stress and altered mitochondrial function, which may influence responsiveness to certain treatments [21, 22]. For example, several studies suggest that immunotherapies might work better in viral-related HCC than in other etiologies of HCC [23].

OXA-based HAIC has shown promising anti-tumor activity for HCC with a high intrahepatic burden and provided a significant survival benefit for patients with advanced HCC [24]. However, the present response rates are still unsatisfactory according to recent reports [10]. Appropriate patient selection is thus of paramount importance and predictive biomarkers for OXA-based HAIC sensitivity are urgently needed. Currently, there are no reports on the efficacy of OXA-based HAIC in NASH-HCC. Here, we show that NASH blunted the effect of OXA-based HAIC therapy against HCC in the survival analysis and murine models of NASH-HCC. Therefore, our findings may have potential clinical implication for the treatment of patients with HCC and with an underlying NASH condition. In the future, extensive work of larger cohorts of OXA-based HAIC treated NASH-HCC patients is needed to verify the rational patient stratification for optimal therapy response.

The lncRNAs, involved in several biological processes, including transcriptional regulation, mRNA splicing, and chromatin modification, are associated with human NASH phenotypes [25]. Their roles in the development of OXA resistance among NASH-HCC patients remain obscure. In this study, lnc-OXAR was first found elevated in both NASH-HCC patients and OXA resistant patients. In cultured cells and NASH mice models, lnc-OXAR confers OXA resistance by stabilizing Ku70, which are able to decrease OXA-induced DSBs.

In general, oxaliplatin induces the formation of inter- and intrastrand DNA-platinum adducts, leading to apoptosis [26]. The tumor cells that lack proper DNA repair cannot cope with the DNA damage and die [27]. Currently, resistance to OXA is a major hurdle for OXA clinical application and one of the popular topics in the context of Pt compounds is mechanisms of resistance. In common human tumors, the mechanism of DNA repair plays a crucial role in developing resistance to chemotherapy. The study of Pt-resistant tumors has provided further convincing evidence, demonstrating the restoration of critical DNA repair pathways. For instance, enhanced binding of Ku70 and Ku80 facilitates nonhomologous end joining (NHEJ), a predominant pathway [28] to promote DSB repair, thereby promoting oxaliplatin chemoresistance in pancreatic cancer [29]. It is now clear that Ku70 participates in DSB repair through NHEJ pathway. However, why Ku70 protein upregulated in NASH-HCC is still unclear. In our study,we showed the interaction between the lnc-OXAR and Ku70.

It was proved that CSTA could be specifically recruited by lnc-OXAR to stabilize Ku70 in this study. Similarly, it was reported that the overexpression of CSTA or CSTB spared ITGB1 from degradation and promoted dissemination of HCC cells [18]. Mechanistically, we have demonstrated that lnc-OXAR participates in OXA resistance by interacting with Ku70 and CSTA to stabilize Ku70 that is essential for starting DSB repair.

In eukaryotic cells, m^6^A modification is the most abundant internal mRNA modification, as an essential role in many physiological and pathological processes, such as carcinogenesis and metabolic dysfunctions [30]. It has been reported that dysregulated m^6^A modification promotes lipogenesis and development of NAFLD and hepatocellular carcinoma [31]. Here we demonstrated that m^6^A modification levels are significantly increased in NASH-HCC tissues, suggesting that increased m^6^A modification levels in total RNA might be an obligate event during the occurrence and development of NASH-HCC. m^6^A modification is a reversible epigenetic modification that exists on mRNAs and non-coding RNAs and controls their fate [32]. It was shown that the long non-coding RNA X-inactive specific transcript (XIST) mediates its gene repression function in a manner dependent on m^6^A modification formation and recognition [33]. In our study, we showed that WTAP-mediated m^6^A modification was significantly enriched in lnc-OXAR. We also revealed a pathway of m^6^A modification formation and recognition required for lnc-OXAR-mediated OXA resistance in NASH-HCC. As a regulatory component of the m^6^A-methyltransferase machine, WTAP functions as a recruiter to drive the METTL3-METTL14 complex to its mRNA or lncRNA targets, and knockdown of WTAP has been shown to protect from diet-induced obesity, leading to improved insulin sensitivity [34]. NASH is frequently associated with obesity, metabolic syndrome [35], which may explain the WTAP upgradation in NASH-HCC patients. It has been reported that m^6^A writer KIAA1429 is a key player in OXA resistance of gastric cancer [36]. Besides, IGF2BP2 has been shown to promote NASH development and may also drive progression from NAFLD to HCC in mouse models [37, 38].

In summary, our study indicated that NASH-HCC benefit less from OXA-based HAIC. We demonstrate that WTAP-mediated lnc-OXAR regulates Ku70 protein post-translationally, resulting in OXA resistance in NASH-HCC (Fig. 6F). These findings provide novel insights into the role of lncRNAs in promoting OXA resistance in NASH-HCC and imply that lnc-OXAR bear the potential to become a novel target for overcoming OXA resistance and improving the prognosis of NASH-HCC.

### Supplementary Information

Additional file 1.

Additional file 2: Supplementary Fig 1. Identification of lnc-OXAR as an OXA resistance–related lncRNA in NASH-HCC. (A) Representative images of tumor burden post-intrahepatic injection (Huh-7) for orthotopic HCC mice fed a control diet or western diet using in vivo bioluminescent imaging. And measurement of tumor burden weekly. (B) HE IHC staining of Ki67 and γH2AX in the tumors. Representative images of four xenografts from each group are shown. Scale bar = 50 μm. (C) Heat maps displaying the RNA-seq profiles and supervised hierarchical clustering analysis for OXA-S and OXA-R HCC patients. Significantly differentially expressed transcripts matching the threshold (more than 2- fold difference) and the statistical analysis standard adjusted P value < 0.05 were selected. (D) Volcano plot shows the differentially express genes identified from RNA-seq analysis of HCC patient samples treated with OXA-based HAIC (OXA-S VS OXA-R). (E) Volcano plot shows the differentially express genes identified from RNA-seq analysis of HCC patient samples (NASH HCC VS Non-NASH HCC). (F) Schematic representation of the generation of OXA-R cell line. (G) The relative viability curves of MHCC-97H cells in OXA-P and OXA-R cells treated with different concentrations of oxaliplatin for 48h. (H) Apoptosis analysis of the apoptotic cells in OXA-P and OXA-R MHCC-97H cells under the treatment of OXA (20 μM, 48h).

Additional file 3: Supplementary Fig 2. lnc-OXAR promoted OXA resistance in NASH HCC. (A) The relative expression of lnc-OXAR in HCC cell lines and MIHA normal human hepatocyte cell line. lnc-OXAR was detected by qPCR and normalized to β-actin. (B) IC50 values of seven HCC cell lines. (C) Scatterplot analyzing the correlation between IC50 values and lnc-OXAR expression levels in the seven HCC cell lines. (D) lnc-OXAR expression in HCC cells transduced with si lnc-OXAR or NC. (E) lnc-OXAR expression in HCC cells transduced with vector or lnc-OXAR OE. (F) The relative viability curves of Huh-7 cells treated with different concentrations of oxaliplatin for 48 h after transfection with siNC and si lnc-OXAR. (G) Cell proliferation was assessed by CCK8 assays in Huh-7 cells transfected with siNC and si lnc-OXAR. (H) Colony formation assays and statistical analysis of Huh-7 cells transduced with siNC or si lnc-OXAR. (I) EdU detection in Huh-7 cells transfected with siNC and si lnc-OXAR with or without OXA treatment. Scale bar = 20μm. (J) The relative viability curves of HepG2 cells treated with different concentrations of oxaliplatin for 48 h after transfection with Vector and lnc-OXAR OE. (K) Cell proliferation was assessed by CCK8 assays in HepG2 cells transfected with Vector and lnc-OXAR OE. (L) Colony formation assays and statistical analysis of HepG2 cells transduced with Vector and lnc-OXAR OE. (M) The effect of lnc-OXAR overexpression on apoptosis in HepG2 cells treated with oxaliplatin (20μM, 48h). (N) EdU detection in HepG2 cells transfected with Vector and lnc-OXAR with or without OXA treatment. Scale bar = 20μm. (O) Representative images of comet assay of HepG2 cells transfected with vector and lnc-OXAR with OXA treatment (20μM, 48h) and quantitative analysis. Scale bar = 20 μm. (P) lnc-OXAR expression in HCC cells transduced with sg lnc-OXAR or NC.

Additional file 4: Supplementary Fig 3. lnc-OXAR promotes OXA resistance in vivo. (A) The effect of lnc-OXAR KO on the tumor growth of subcutaneously implanted PLC/PRF/5 cells treated with OXA (10 mg/kg) or vehicle control in nude mice (*n*=5). Scale bars, 1 cm. The tumor weights at the end points and measurement of tumor volumes weekly. GS, glucose saline. (B) The effect of lnc-OXAR KO on the tumor growth of subcutaneously implanted Huh-7 cells treated with OXA (10 mg/kg) or vehicle control in nude mice (*n*=5). Scale bars=1 cm. The tumor weights at the end points and measurement of tumor volumes weekly. (C) The effect of lnc-OXAR OE on the tumor growth of subcutaneously implanted Huh-7 cells treated with OXA (10 mg/kg) or vehicle control in nude mice (*n*=5). Scale bars=1 cm. The tumor weights at the end points and measurement of tumor volumes weekly.

Additional file 5: Supplementary Fig 4. lnc-OXAR maintained Ku70 stability by recruiting CSTA. (A) Enriched GO pathways of lnc-OXAR-binding proteins screened by ChIRP-MS assays (unique peptide ≥2, fold change >1.5). (B) The mRNA level of Ku70 between peritumor and tumor samples in TCGA cohort. (C) The IHC score of Ku70 in paired peritumor and tumor samples (*n*=228 pairs) in SYSUCC cohort. (D) Overall Survival rate of HCC patients categorized according to median Ku70 expression in TCGA cohort. (E) OS rate of HCC patients categorized according to median Ku70 expression in SYSUCC cohort. (F) RFS rate of HCC patients categorized according to median Ku70 expression in SYSUCC cohort. (G) The distribution of AFP (<400, ≥400) determined by the IHC score of Ku70 in the high or low groups in SYSUCC cohort. p values were determined by two-tailed Chi-square test. (H) The distribution of stage (I-II, III-IV) determined by the IHC score of Ku70 in the high or low groups in SYSUCC cohort. (I) Representative IHC staining images of Ku70 expression HCC tissues. Scale bar = 100 μm. (J) Ku70 RIP-qPCR analysis of lnc-OXAR level in Huh-7 cells. (K) Ku70 RIP-qPCR analysis of lnc-OXAR level in HepG2 cells. (L) Ku70 mRNA level in HCC cells after lnc-OXAR KO. (M) WB showing Ku70 protein in Huh-7 cells with or without lnc-OXAR KO treated with CHX for the indicated time. (N) WB showing Ku70 protein in HepG2 cells with or without lnc-OXAR OE treated with CHX for the indicated time. (O) CSTA RIP-qPCR analysis of lnc-OXAR level in Huh-7 and HepG2 cells. (P) WB results verified that Ku70 associated CSTA. (Q) WB of immunoprecipitated Ku70 and CSTA to determine the effect of lnc-OXAR8 KO on Huh-7 cells. (R) &(S) IF staining showed the co-localization of CSTA (green) and Ku70 (red) in HCC cells. Scale bar = 10 μm. (T) WB showing the effect of CSTA silencing on Ku70 protein in HepG2 cells with or without lnc-OXAR OE after treated with CHX for the indicated time.

Additional file 6: Supplementary Figure 5. The effect of lnc-OXAR on OXA resistance is dependent on Ku70. (A) Validation of Ku70 KD in PLC/PRF/5 cells. (B) The effect of Ku70 KD on the apoptosis of HCC cells with treatment (20 μM,48h) using flow cytometry analysis of Annexin V staining. (C) The effect of Ku70 OE on the CCK8 assay of lnc-OXAR KO in OXA-R cells without OXA treatment. (D) The effect of Ku70 KD on the CCK8 assay of lnc-OXAR OE in OXA-R cells without OXA treatment. (E) Images of colony formation assays in HCC cells with or without OXA treatment. (F) The effect of Ku70 OE on the apoptosis of lnc-OXAR-KO in OXA-R cells with treatment (20 μM,48h) using flow cytometry analysis of Annexin V staining. (G) The effect of Ku70 KD on the apoptosis of lnc-OXAR-overexpressing in HepG2 cells with treatment (20μM,48h) using flow cytometry analysis of Annexin V staining. (H) The effect of Ku70 OE on the colony-formation of lnc-OXAR KO in OXA-R cells with or without OXA treatment. (I) The effect of Ku70 KD on the colony-formation of lnc-OXAR-overexpressing in HepG2 cells with or without OXA treatment. (J) The effect of Ku70 KD on the EDU assay of lnc-OXAR-overexpressing in HepG2 cells without OXA treatment. Scale bar = 50 μm.

Additional file 7: Supplementary Fig 6. The effect of lnc-OXAR on OXA resistance is dependent on Ku70. (A) Validation of Ku70 KD in PLC/PRF/5 cells. (B) HE staining and IHC staining of Ki67, Ku70 and γH2AX in the tumors. Representative images of four xenografts from each group are shown. Scale bar = 50 μm.

Additional file 8: Supplementary Fig 7. m6A modification mediated lnc-OXAR upregulation in NASH-HCC. (A) lnc-OXAR expression level was detected after treated with 5-AZA in PLC/PRF/5 and Huh-7 cells. (B) lnc-OXAR expression level was detected after treated with TSA in PLC/PRF/5 and Huh-7 cells. (C) lnc-OXAR expression in HCC cells treated with 50 μM DAA or vehicle. (D) mRNA validation of WTAP knockdown in HCC cells. (E) The degradation rate of lnc-OXAR after WTAP silencing in PLC/PRF/5 cells with actinomycin D. (F) The degradation rate of lnc-OXAR after WTAP silencing in Huh-7 cells with actinomycin D. (G) The degradation rate of lnc-OXAR after WTAP silencing in HepG2 cells with actinomycin D. (H) Correlation between lnc-OXAR and WTAP expression in HCC patients from SYSUCC. (I) mRNA validation of six m6A readers’ knockdown in HCC cells. (J) The degradation rate of lnc-OXAR after IGF2BP2 silencing in PLC/PRF/5 cells with actinomycin D. (K) Degradation rate of lnc-OXAR after IGF2BP2 silencing in Huh-7 cells with actinomycin D. (L) Degradation rate of lnc-OXAR after IGF2BP2 silencing in HepG2 cells with actinomycin D. (M) Correlation between lnc-OXAR and IGF2BP2 expression in HCC patients from SYSUCC.

## Data Availability

The datasets used and/or analyzed during the current study are available from the corresponding author on reasonable request.

## Abbreviations

HCC: Hepatocellular carcinoma
NASH: Non-alcoholic steatohepatitis
OXA: Oxaliplatin
lncRNA: Long non-coding RNA
lnc-OXAR: OXA resistance–related lncRNA in NASH-HCC
DSB: DNA double-strand break
m^6^A: N6-Methyladenosine
WTAP: WT1 Associated Protein
IGF2BP2: Insulin Like Growth Factor 2 MRNA Binding Protein 2
CSTA: Cystatin A
NAFLD: Non-alcoholic fatty liver disease
HAIC: Hepatic arterial infusion chemotherapy
ChIRP-MS: Comprehensive identification of RNA-binding proteins by mass spectrometry
PDX: Patient-Derived tumor Xenograft
