# Supplementary material for: m6A-mediated lnc-OXAR promotes oxaliplatin resistance by enhancing Ku70 stability in non-alcoholic steatohepatitis-related hepatocellular carcinoma
Source: J Exp Clin Cancer Res. 2024 Jul 25;43:206. doi: 10.1186/s13046-024-03134-4 (PMC11271202; doi:10.1186/s13046-024-03134-4)
Supplement: Supplementary file 1 — Additional file 1. [file 13046_2024_3134_MOESM1_ESM.docx]

**Supplementary Materials and Methods**

**Cell Lines and cell culture**

Human HCC cell lines (PLC/PRF/5, Huh7, Hep3B, and HepG2) were purchased from the Shanghai Cell Bank of the Chinese Academy of Sciences (Shanghai, China) and MHCC-97H was purchased from Jennio Biotech with STR (short tandem repeat) appraisal certificates. Cells were maintained in Dulbecco’s Modified Eagle medium (DMEM; ThermoFisher, USA) supplemented with 10% fetal bovine serum (FBS; Gibco, California, USA) at 37 °C in 5% CO2.

**Subcellular fractionation for RT-qPCR.**

A Cytoplasmic & Nuclear RNA Purification Kit (Norgen Biotek Corp, Canada) was used to detect lnc-OXAR expression in cytoplasmic and nuclear fractions. According to the manufacturer’s instructions, RNA was extracted from the cytoplasmic and nuclear fractions and subjected to qPCR. β-Actin was used as a cytoplasmic marker, and U6 was used as a nuclear marker.

**RNA pull-down and RIP assays.**

RNA was transcribed in vitro using a MEGA script T7 Transcription Kit (Invitrogen) and biotinylated using a Pierce RNA 3’ End Desthiobiotinylation Kit (Thermo Scientific) according to the manufacturers’ instructions. Cells were prepared using Pierce IP lysis buffer (Thermo Scientific). RNA pull-down assays were performed with a Pierce Magnetic RNA–Protein Pull Down Kit. According to the manufacturer’s instructions, biotinylated RNA was captured with streptavidin magnetic beads and then incubated with cell lysates or purified protein (20 µg) at 4 °C for 6 h before washing and elution of the RBP complex. The eluted proteins were subjected to MS analysis or western blotting. RIP assays were performed with a Magna RNA-binding protein immunoprecipitation kit (Millipore, Bedford, MA) according to the manufacturer’s instructions. Negative control IgG, human anti-Ku70 (1:20, Abcam, ab181861), anti-WTAP (1:20, Cell Signaling Technology, 8146), anti-IGF2BP2 (1:20, Cell Signaling Technology, 8146) and anyi-CSTA (1:20, Cell Signaling Technology, 8146) bodies were used in this study. After proteinase K digestion, the immunoprecipitated RNAs were extracted, purified, and subjected to qPCR. RNA levels were normalized to the input (10%).

**Stable cell line construction and RNA interfering**

lnc-OXAR KO cell lines were generated using a CRISPR/Cas9-based strategy(39). lnc-OXAR-specific guide RNA (gRNA) expression sequencing primer GGCCATCCTGCGTTTCCGCC were used to detect the efficiency of lnc-OXAR sgRNA. Thus, double sgRNA vectors were constructed using the sgRNAs mentioned above. After the construction of the vectors, the vectors were sequenced and compared with the target genes, which showed that the vectors were constructed correctly. HCC cells were transfected with the pLV-U6-lnc-OXAR RNA sgRNA01-7SK-sgRNA02-EFS-hCas9-2A-Puro or pLV-U6-NC sgRNA01-7SK-NC sgRNA02-EFS-hCas9-2A-Puro expression vectors. The transfected cells were selected using puromycin (5 µg per ml) for 7 days. Isolated single colonies were expanded and subjected to detection of genomic deletions by PCR. Small interfering RNA for Ku70 were purchased from Genepharma (Shanghai, China). Reverse transfection of small interfering RNA was performed with Lipofectamine-3000 (Invitrogen, Carlsbad, CA). After 24 hours, the supernatant was replaced with fresh medium and the down-regulation efficiency was identified by qRT-PCR and western blotting 48 hours after co-transfection.

**Cell proliferation, colony formation and apoptosis assays**

For the cell proliferation assay, 1000 cells were seeded into 96-well plates, cell viability was assessed for 5 consecutive days with or without oxaliplatin treatment (1umol/L) by the Cell Counting Kit-8 (CCK-8) (Dojindo, Japan). For the colony formation assay, 1000 cells were seeded into 6-well plates for 2 weeks with or without oxaliplatin treatment (0.5umol/L), and colonies were stained with crystal violet and counted. All studies were conducted in triplicates. For the apoptosis assay, cells were treated with oxaliplatin (40umol/L) for 48h pretreated with either Annexin V/APC or 7-AAD (KeyGEN BioTECH, China) according to the manufacturer’s instructions.

**RNA extraction and quantitative real-time PCR**

Total RNA was isolated from cell lines using RNA Quick Purification kit (ESscience Cat # RN001) according to the manufacturer’s instructions. Complementary DNA was synthesized using random hexamers and superscript III (Invitrogen, California, USA) from 1µg of total RNA. Then, quantitative real-time PCR was performed on LightCycler 480 II detector (Roche, Basel, Switzerland) using SYBR Green PCR kit (Invitrogen, California, USA). All experiments were run in triplicate, and β-actin was used as control gene to normalize the gene expression levels.

**Western blotting assays**

The extracted whole cell protein lysates were prepared with cell lysis buffer (Cell Signaling Technology, Boston, USA) supplemented with protease inhibitor cocktail (Roche, Basel, Switzerland) and phosphatase inhibitor cocktail (Roche, Basel, Switzerland), when phosphorylated proteins were needed to detect. Then, the equal amounts of protein lysates were separated by SDS-polyacrylamide gels and transferred to polyvinylidene difluoride membranes (Merck Millipore, Cork, Ireland). After incubated with the indicated primary and secondary antibodies the bindings were detected using High-sig ECL western blotting kit (Tanon, shanghai, China).

**Comet assay**

Comet assay was analyzed using DNA Damage Detection Kit (KeyGen BioTech, Nanjing) according to the manufacturer’s instructions. Briefly, transfected cells were treated with OXA for 48h. Cells were collected and suspended in PBS containing 1% low-melting agarose and layered onto adhesive microscope slides previously covered with 0.5% normal-melting agarose. The cells were dipped in a specific lysed buffer at 4°C for 2 h. Then, the DNA was uncoiled and unwound in an alkalescent electrophoresis buffer for 30 min. Electrophoresis was carried out and the cells were stained with DAPI solution for 10 min in a dark room. The slides were examined with an Olympus BX63 fluorescence microscope. Tail moment was calculated by using CASP software.

**Immunohistochemistry (IHC)**

For immunohistochemistry analysis, tissue specimen slides were deparaffinized, rehydrated through an alcohol series followed by antigen retrieval with sodium citrate buffer.

Tumor sections were blocked with 5% normal goat serum (Vector) with 0.1% Triton X-100 and 3% H2O2 in PBS for 60 min at room temperature and then incubated with appropriate primary antibodies 4°C overnight. IHC staining was performed with horseradish peroxidase (HRP) conjugates using DAB detection. Nuclei were counterstained with Hoechst. Images were taken with Nikon microscopy.

For IHC scoring, two experienced pathologists evaluated the staining intensity of specific markers, independently. The immunoreactivity for S100A9 protein was scored using a semi-quantitative method by evaluating the number of positive tumor cells over the total number of tumor cells, and the IHC scores were assigned by using 5% increments (0, 5, 10%...100%), as described in previous studies(40, 41). Based on the IHC scores, we then dichotomized these patients as negative group, 0–25%; weak group, 25–50%; moderate group, 50–75%; strong group, 75–100%.

**Luciferase reporter assay**

Luciferase activity was measured by the Dual-luciferase Reporter Assay System (Promega, Madison, USA) according to the manuscript’s instructions. Briefly, HCC cells were co-transfected with each Firefly luciferase reporter construct in combination with the Renilla luciferase construct, and both luciferase activities in cell extracts at 48h after transfection were measured using a Steady-Glo® Luciferase Assay System (Promega). The Firefly luciferase activity was normalized to the Renilla luciferase activity that reflects expression efficiency.

**Dot blotting assay**

First, isolated mRNA (200 ng for each group) was denatured by heating at 95 °C for 3 min, followed by chilling on ice immediately. Dilutions were spotted on an Amersham Hybond-N +membrane optimized for nucleic acid transfer (GE Healthcare). The membrane was crosslinked under UV light and washed with 1× PBST buffer. After the membrane was blocked with 5% nonfat milk in PBST, it was incubated with anti-m6A antibody (202003, Synaptic Systems) overnight at 4 °C. Then, the membrane was incubated with secondary antibody at room temperature for 1 h. Immunoblots were developed using a chemiluminescent reagent (Beyotime). The same 200 ng of mRNAs were spotted on the membrane, stained with 0.02% methylene blue in 0.3 M sodium acetate (pH 5.2) for 2 h and washed with RNase-free water for 1 h.

**RNA stability assay**

HCC cell lines were plated in 6-well plates and exposed to actinomycin D (5 μg/mL, Sigma) for 0, 3, and 6 h. RNA was extracted at the indicated times and analyzed by qPCR. We calculated the mRNA half-life value by linear regression analysis.

**MeRIP (m^6^A)-seq and RNA-immunoprecipitation seq, RIP-qPCR (RIP-qPCR)**

For meRIP-seq, total RNA in OXA-S and OXA-R cells was extracted by using TRIzol™ Reagent (Invitrogen™, Cat# 15596018) and detected by Bioptic Qseq100 Bio-Fragment Analyzer (Bioptic lnc.). DNase I (Invitrogen™, Cat# EN0525) treatment was adopted to remove DNA contamination. Additional phenol-chloroform isolation and ethanol precipitation treatments were performed to remove enzyme ontamination. For meRIP-Seq, 20μg purified RNA was fragmented into ~200 nucleotide-long fragments by incubating in magnesium RNA fragmentation buffer for 6 min at 70℃. The fragmentation was stopped by adding EDTA. Then, Zymo RNA Clean and Concentrator-5 Kit was used to purify fragmented total RNA (Zymo Research™, Cat# R1013). Next, m6A immunoprecipitation was performed by using EpiTM m6A immunoprecipitation kit (Epibiotek™, Cat# R1804). Breifly, protein A magnetic beads ((Invitrogen™, Cat# 10002D), protein G magnetic beads ((Invitrogen™, Cat# 10004D) and anti-N6-methyladenosine (m6A) Antibody（ Sigma-Aldric™, Cat#ABE572） were mixed together and incubated at 4℃ overnight. After the beads-antibody incubation, the beads were recovered by magnet and resuspended within 5X precipitation buffer solution and RNase Inhibitor and incubated at 4℃ for another 2h. The beads-antibody-RNA mixture was washed twice with high-salt buffer and twice with low-salt IP buffer. After extensive washing, bound RNA was eluted from the beads with wash buffer solution, then additional phenol-chloroform isolation and ethanol precipitation treatment were performed to purify the bound RNA .For RIP- Seq, cells were collected and then the pellet was resuspended in lysis buffer and rotated for 30min at 4℃. After cell lysis, harvested the lysate by centrifugation at 12000g for 10min. Transfer the supernatant into a fresh 1.5ml tube. Note that adding protease inhibitor and RNase inhibitor into the lysis buffer. Keep about 10% volume of lysate and exacted the RNA as the input to detecting the RNA integrity. The followed RIP steps were performed by using EpiTM RNA immunoprecipitation kit (EpibiotekTM, Cat#R1819). 40μl of protein G beads was washed twice by IP buffer and added into the lysate together with antibody, followed by incubation overnight at 4℃. After incubation, transfer the supernatant into a fresh 1.5ml tube. Recover the beads by magnet and resuspended within 1× wash buffer, rotated at 4℃ for 10min. Remove the supernatant and repeat the washing step for three times. Extracted the co-precipitated RNA by TRIzol™ Reagent (Invitrogen™, Cat# 15596018) and Phenol-chloroform method. Co-precipitated RNA and input RNA were subjected to library construction by using EpiTM mini longRNA-seq kit (Epibiotek, Cat# E1802) according to the manufacturer’s protocols. Briefly, reverse transcription was performed using random primers and the ribosome cDNA (cDNA fragments originating from rRNA molecules) was removed after cDNA synthesis using probes specific to mammalian rRNA. The directionality of the template-switching reaction not only preserves the 5’ end sequence information of RNA but the strand orientation of the original RNA. Libraries for immunoprecipitated RNA were PCR amplified for 18 cycles. Library quality was determined using Qseq100 Bio-Fragment Analyzer（ Bioptic lnc.). The strand-specific libraries were sequenced on Illumina Novaseq 6000 system with paired-end 2×150 bp read length.

**IF staining and FISH**

Cells grown in 8-well BD Falcon cell culture slides (Millipore, MI, USA) were washed three times with ice-cold PBS and then were fixed with 4% paraformaldehyde (Jetway Biotech, Guangzhou, China) for 15 min. After permeabilizing with 0.2% Triton X-100 for 10 min at room temperature, the cells were blocked in goat serum (Zsbio, Beijing, China). Thereafter, the cells were incubated with anti-Ku70 or anti-CSTA antibodies, at 4℃ overnight. Then, after washing with ice-cold PBS for three times, the cells were incubated with AlexaFluor488 or AlexaFluor594 goat anti-rabbit IgG (Invitrogen, California, USA) at room temperature for 1h avoiding lights. The nuclei staining was performed by DAPI (Beyotime, Shanghai, China) for 10 min, and then the stained cells were observed with a laser scanning confocal microscope.

FISH assays were carried out with a lncRNA FISH Kit (RiboBio, Guangzhou, China). In brief, cells were fixed and permeabilized in PBS containing 0.5% Triton X-100. FISH probes were designed by RiboBio (Guangzhou, China). Hybridization was carried out overnight in a humidified chamber at 37 °C in the dark. All images were obtained with an Olympus FV1000 confocal microscope (Tokyo, Japan). 4′,6-diamidino-2-phenylindole and Cy3 channels were used to detect the signals.

IF combined with FISH (IF-FISH) was performed as previously described with minor modifications. Briefly, after being incubated with secondary antibodies, cover slips were washed and fixed with 4% PFA for 10 min, dehydrated in 70%, 85%, and 100% ethanol, and air dried. Samples were denatured at 85 °C for 5 min in presence of Cy3-or Cy5-labeled (CCCTAA)4 PNA probes (TelC) (Panagene) or XMP mouse chromosome 5 or 8 painting probes (Metasystems). Hybridization was then carried out at 37 °C for >12 h. Cover slips were washed once with hybridization buffer, twice with 2×SSC buffer, and once with PBS supplemented with 1 μg/mL DAPI. Cover slips were then air-dried and ready for imaging. Microscopy imaging was performed by LSM 880 (Zeiss) using ×60NA/1.40 oil.

**Comprehensive Identification of RNA-Binding Proteins by Mass Spectrometry (ChIRP-MS)**

The antisense oligo probes of lnc-OXAR were designed, synthesized, and modified with biotin-TEG at the 3′ ends by KangChen Bio-tech (Shanghai, China). HK-2 cells (1 × 108 cells per sample) were then crosslinked with 3% formaldehyde solution at 25°C for 10 min. Lysis buffer was added to the solution and was centrifuged to remove the supernatant. Next, two volumes of the hybridization buffer were added and mixed. This was combined with the biotin-labelled probe and the magnetic beads for 30 min. After overnight hybridization at 37°C, elution buffer (100 μl) was added to resuspend the magnetic beads, and nuclease benzonase was added to react at 37°C for 1 h. After further reaction at 95°C for 30 min, the suspension was crosslinked with 0.1% SDC and 10% TCA and precipitated at 4°C for 2 h. ABC (200 ml; 100 mM, pH 8.5, 0.1% SDC) was added to dissolve the precipitate, and 5 mm TCEP, 10 mM IAA, 0.5 μg Trypsin, and TFA were subsequently added to terminate the digestion. Precipitation was desalinated for MS detection. The enzymatic hydrolysates were separated using nano-UPLC and analysed online using a Q-Exactive mass spectrometer (Thermo Finnigan). The MS raw data were quantitatively analysed using MaxQuant.

**Table S2. Patient characteristics.**

| Variables | Non-NASH HCC | NASH-HCC | *P* value |
| --- | --- | --- | --- |
| Age (years) |  |  |  |
| ≥ 52 | 33 | 35 | 0.0704 |
| < 52 | 17 | 15 |  |
| Sex |  |  |  |
| Male | 43 | 41 | 0.0374 |
| Female | 7 | 9 |  |
| Liver cirrhosis |  |  |  |
| Yes | 31 | 27 | 0.0489 |
| No | 19 | 13 |  |
| HBsAg |  |  |  |
| Positive | 41 | 36 | 0.1166 |
| Negative | 9 | 14 |  |
| Tumor size (diameter, cm) | | | |
| ≤ 5 | 36 | 32 | 0.1392 |
| > 5 | 14 | 18 |  |
| Tumor number |  |  |  |
| Multiple | 12 | 17 | 0.1488 |
| Single | 38 | 33 |  |
| Microvascular invasion | | | |
| Yes | 15 | 17 | 0.0704 |
| No | 35 | 33 |  |
| BCLC stage |  |  |  |
| A | 5 | 7 | 0.0903 |
| B | 24 | 21 |  |
| C | 21 | 22 |  |

**Table S2.** **Information on lnc-OXAR-binding proteins screened by ChIRP-MS assay in the present study.**

| Gene Symbol | Unique peptides | Sequence coverage | Score | MS count ctrl | MS count Lnc | iBAQ | fc.Lnc-Ctrl |
| --- | --- | --- | --- | --- | --- | --- | --- |
| ENO1 | 5 | 59.2 | 53.27 | 0 | 2 | 2198700 | 2.293536494 |
| ALOX12B | 6 | 19.6 | 37.189 | 5 | 9 | 15289000 | 2.293536494 |
| GSDMA | 5 | 8 | 29.956 | 0 | 3 | 489550 | 2.293536494 |
| NCCRP1 | 5 | 37.3 | 30.802 | 1 | 5 | 511930 | 1.973399338 |
| SBSN | 4 | 12 | 29.7 | 1 | 3 | 5196900 | 1.973399338 |
| LTF | 6 | 24.2 | 83.833 | 2 | 4 | 1684200 | 1.973399338 |
| CALML5 | 9 | 15.8 | 61.216 | 4 | 9 | 1220000 | 1.973399338 |
| XRCC6 | 4 | 20.5 | 27.928 | 3 | 9 | 961240 | 1.973399338 |
| ALOXE3 | 2 | 19.5 | 15.952 | 0 | 2 | 6876100 | 1.973399338 |
| RPL18 | 2 | 3.2 | 13.077 | 0 | 2 | 642270 | 1.973399338 |
| S100A8 | 4 | 29.3 | 27.106 | 0 | 2 | 91186 | 1.973399338 |
| S100A9 | 5 | 47.3 | 49.218 | 0 | 3 | 1387200 | 1.973399338 |
| SERPINB3 | 4 | 37.7 | 31.099 | 4 | 8 | 13617000 | 1.973399338 |
| SPRR1B | 2 | 9.2 | 21.519 | 3 | 7 | 9853100 | 1.973399338 |
| PRDX2 | 4 | 61.8 | 27.785 | 0 | 3 | 521350 | 1.973399338 |
| TGM1 | 3 | 18.7 | 16.592 | 4 | 7 | 14357000 | 1.973399338 |
| POF1B | 13 | 17.5 | 88.297 | 1 | 4 | 3816900 | 1.973399338 |
| CSTA | 4 | 7.1 | 40.802 | 7 | 13 | 2399400 | 1.973399338 |
| HIST2H2AC | 2 | 16.5 | 22.894 | 1 | 4 | 680590 | 1.973399338 |
| HIST1H4A | 6 | 57.3 | 45.245 | 3 | 7 | 29122000 | 1.973399338 |
| HSPA6 | 5 | 31.4 | 43.856 | 1 | 11 | 221610 | 1.973399338 |
| ALG13 | 4 | 29.8 | 37.618 | 1 | 9 | 33335000 | 1.973399338 |
| HNRNPK | 6 | 20.1 | 36.307 | 1 | 13 | 3239700 | 1.973399338 |
| HIST1H1C | 6 | 28.1 | 35.238 | 1 | 8 | 958610 | 1.704985433 |
| TMEM33 | 3 | 32.7 | 34.979 | 2 | 7 | 956090 | 1.667630158 |
| HSPA8 | 2 | 18.7 | 33.17 | 2 | 5 | 557890 | 1.667630158 |
| SFN | 6 | 38.2 | 31.38 | 2 | 5 | 290980 | 1.667630158 |
| SYPL1 | 4 | 33.3 | 31.051 | 2 | 10 | 193190 | 1.667630158 |
| HSPB1 | 4 | 20.1 | 29.321 | 3 | 7 | 65559 | 1.667630158 |
| ANXA2 | 1 | 26 | 17.411 | 0 | 4 | 4843900 | 1.667630158 |
| LYZ | 2 | 49.9 | 16.086 | 1 | 7 | 10690000 | 1.667630158 |
| LGALS7 | 3 | 37.7 | 51.745 | 1 | 4 | 2626200 | 1.667630158 |
| LGALS7 | 13 | 9.8 | 14.896 | 1 | 5 | 2626200 | 1.667630158 |
| UBC | 7 | 31.9 | 14.534 | 1 | 4 | 1423800 | 1.667630158 |
| CDC5L | 13 | 9 | 23.719 | 4 | 9 | 452510 | 1.667630158 |
| TPI1 | 6 | 90.3 | 14.391 | 2 | 4 | 377960 | 1.667630158 |
| CASP14 | 11 | 28.2 | 51.976 | 3 | 9 | 5839900 | 1.667630158 |
| PSMB5 | 10 | 28.7 | 51.814 | 3 | 8 | 5339700 | 1.667630158 |
| KPRP | 10 | 16 | 51.745 | 0 | 2 | 25068000 | 1.561341804 |
| CST6 | 2 | 25.6 | 28.988 | 6 | 10 | 2510600 | 1.561341804 |
| CTSD | 5 | 56.8 | 27.681 | 3 | 5 | 1320500 | 1.561341804 |
| ALDOA | 4 | 19.9 | 25.241 | 3 | 7 | 307390 | 1.561341804 |
| PKM | 5 | 25.8 | 25.193 | 3 | 8 | 188180 | 1.561341804 |
| PRPF19 | 4 | 30.4 | 25.193 | 4 | 7 | 182040 | 1.561341804 |
| DSC3 | 4 | 18.8 | 24.915 | 3 | 7 | 130200 | 1.561341804 |
| HSPA5 | 4 | 30 | 23.509 | 3 | 6 | 116040 | 1.561341804 |
| SERPINB12 | 3 | 53.1 | 21.757 | 0 | 4 | 4186500 | 1.561341804 |
| S100A14 | 3 | 44.2 | 21.545 | 3 | 12 | 4088700 | 1.561341804 |
| PIP | 4 | 13.7 | 19.42 | 4 | 10 | 23529000 | 1.561341804 |
| PKP1 | 3 | 46 | 18.557 | 4 | 6 | 1631700 | 1.561341804 |

**Table S3. Antibodies included in the study.**

| **Antibodies** | **SOURCE** | **IDENTIFIER** |
| --- | --- | --- |
| Anti-p-H2AX(Ser139) (for IB, IF) | ab81299 | Abcam |
| Anti-β-actin | 66009-1-Ig | Proteintech |
| Anti-Ku70 (for IB, IHC, IF, IP) | 10723-1-AP | Proteintech |
| Anti-Ku70 (for IB, IHC, IF, IP) | 66607-1-Ig | Proteintech |
| Anti-CSTA (for IB, IHC, IF, IP) | sc-376759 | Santa Cruz Biotechnology |
| Anti-WTAP (for IB, IHC, IP, RIP) | 10200-1-AP | Proteintech |
| Anti-IGF2BP2(for IB, IHC, IP, RIP) | 11601-1-AP | Proteintech |
| Anti-Ki67 (for IHC) | ab279653 | Abcam |
| Alexa Fluor 488 | # A-11008 | Thermo Fisher Scientific |
| Alexa Fluor 594 | # R37117 | Thermo Fisher Scientific |
| Anti-mouse IgG | 7076S | Cell Signaling TECHNOLOGY |
| Anti-rabbit IgG | 7074S | Cell Signaling TECHNOLOGY |
| HRP RABBIT/MOUSE | - | DAKO |

**Table S4. The primers used in present study.**

| **Primer names** | **Forward (5’-3’)** | **Reverse (5’-3’)** |
| --- | --- | --- |
| lnc-OXAR | GAGAAACAGGATGTGAGAAATCG | TCTGCTTCATTATCTCACGCTCT |
| Lnc-NP1-5 | ATTGGTGGATTTATGGTGCG | TGAGGTCTTGTGTCGTCGGT |
| LINC00662 | CTGAGCACAGCAGAATACAGGA | TAGTAAGAGGGTGGTAGCAAGGA |
| ACTB | CACCATTGGCAATGAGCGGTTC | AGGTCTTTGCGGATGTCCACGT |
| WTAP | GCAACAACAGCAGGAGTCTGCA | CTGCTGGACTTGCTTGAGGTAC |
| Mettl3 | CTATCTCCTGGCACTCGCAAGA | GCTTGAACCGTGCAACCACATC |
| Mettl14 | CTGAAAGTGCCGACAGCATTGG | CTCTCCTTCATCCAGATACTTACG |
| ALKBH5 | CCAGCTATGCTTCAGATCGCCT | GGTTCTCTTCCTTGTCCATCTCC |
| FTO | CCAGAACCTGAGGAGAGAATGG | CGATGTCTGTGAGGTCAAACGG |
| IGF2BP1 | CTTTGTAGGGCGTCTCATTGGC | CCTTCACAGTGATGGTCCTCTC |
| IGF2BP2 | GTTGGTGCCATCATCGGAAAGG | TGGATGGTGACAGGCTTCTCTG |
| IGF2BP3 | TCGTGACCAGACACCTGATGAG | GGTGCTGCTTTACCTGAGTCAG |
| YTHDC2 | GAAAGCTCCTGAACCTCCACCA | GGTTCTACTGGCAAGTCAGCCA |
| YTHDF2 | TAGCCAGCTACAAGCACACCAC | CAACCGTTGCTGCAGTCTGTGT |
| YTHDF3 | GCTACTTTCAAGCATACCACCTC | ACAGGACATCTTCATACGGTTATTG |
| U6 | CTCGCTTCGGCAGCACAT | TTTGCGTGTCATCCTTGCG |
| lnc-OXAR(for MeRIP-qPCR) | AGGGGATGTGTGTGAGAAGG | GCTTCGAGACAGTGGGAGTC |

**table S5. Sequences of lncRNA smart silencers.**

| **Gene symbol** | **siRNAs** | **ASOs** |
| --- | --- | --- |
| lnc-OXAR | CAGGATGTGAGAAATCGAGA | CGTGAGATAATGAAGCAGA |
|  | GAGCGTGAGATAATGAAGCA | GAAAGACAAGGACATTGTC |
|  | AAGAGAGAGCGTGAGATAAT | GATGTGAGAAATCGAGACT |
| Negative control | LncRNA smart silencer used as negative control for *lnc-OXAR* does not target any human genomic locus. | |

**Table S6. Sequences of RNA Oligonucleotides.**

| **Name** | **Sense strand/sense primer (5’-3’)** | **Antisense strand/antisense primer (5’-3’)** |
| --- | --- | --- |
| **siRNA** | | |
| siWTAP | GCGAAGUGUCGAAUGCUUATT | UAAGCAUUCGACACUUCGCTT |
| siIGF2BP2 | CAUGCCGCAUGAUUCUUGATT | UCAAGAAUCAUGCGGCAUGTT |
| siCSTA | GGUUAAACCACAGCUUGAATT | UUCAAGCUGUGGUUUAACCTT |
| **shRNA** | | |
| shKu70#1 | GATGAGTCATAAGAGGATCAT | ATGATCCTCTTATGACTCATC |
| shKu70#2 | CGTCAGATTATACTGGAGAAA | TTTCTCCAGTATAATCTGACG |
| **sgRNA** | | |
| sglnc-OXAR | caccGGCCATCCTGCGTTTCCGCC | aaacGGCGGAAACGCAGGATGGCC |
| sgNC | GACCGGGGCGAGGAGCTGTTCACCG | CGGTGAACAGCTCCTCGCCCCGGTC |
