# Supplementary figures and images for: m6A-mediated lnc-OXAR promotes oxaliplatin resistance by enhancing Ku70 stability in non-alcoholic steatohepatitis-related hepatocellular carcinoma
Source: J Exp Clin Cancer Res. 2024 Jul 25;43:206. doi: 10.1186/s13046-024-03134-4 (PMC11271202; doi:10.1186/s13046-024-03134-4)

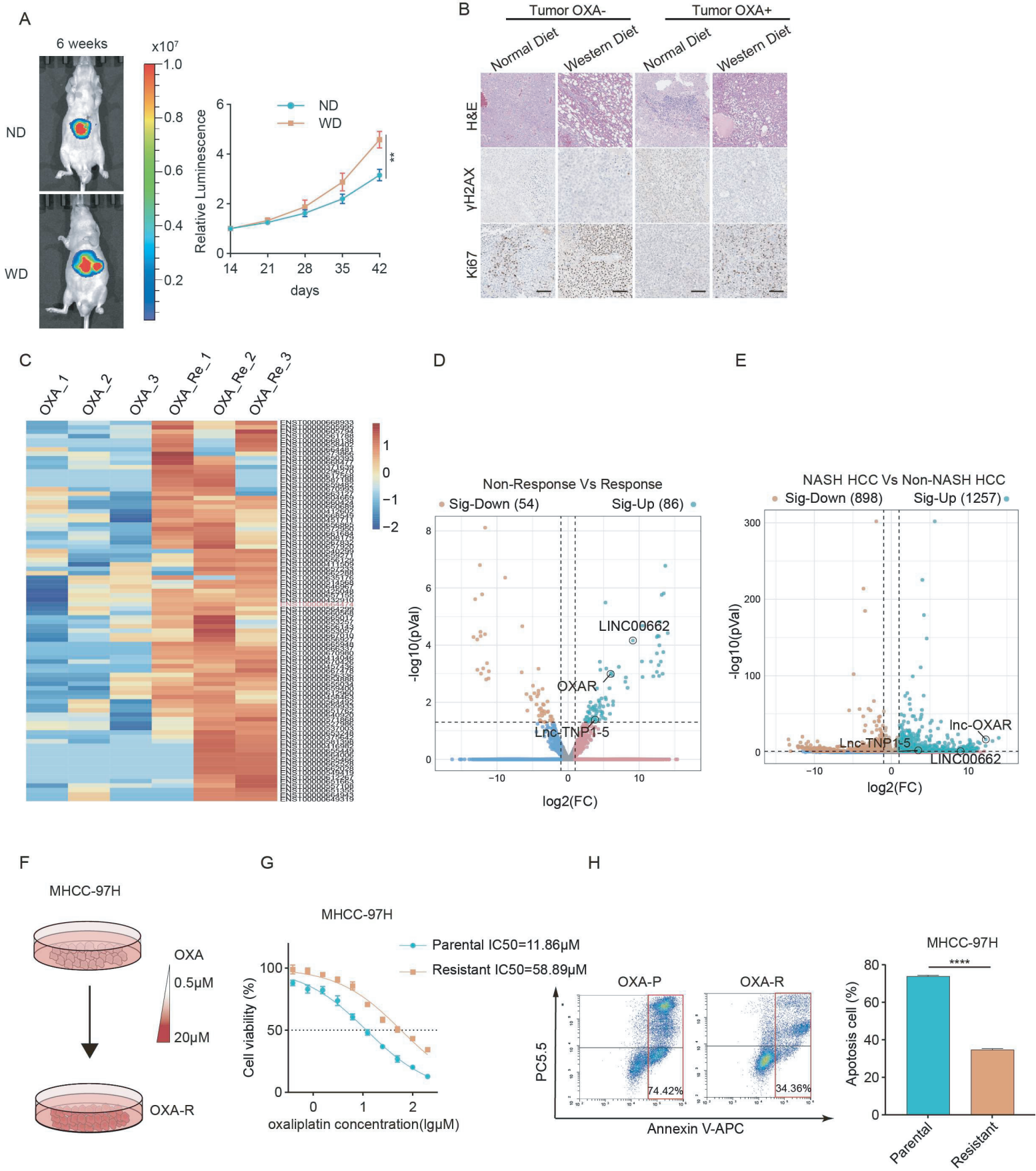

Supplement: Supplementary file 2 — Additional file 2: Supplementary Fig 1. Identification of lnc-OXAR as an OXA resistance–related lncRNA in NASH-HCC. (A) Representative images of tumor burden post-intrahepatic injection (Huh-7) for orthotopic HCC mice fed a control diet or western diet using in vivo bioluminescent imaging. And measurement of tumor burden weekly. (B) HE IHC staining of Ki67 and γH2AX in the tumors. Representative images of four xenografts from each group are shown. Scale bar = 50 μm. (C) Heat maps displaying the RNA-seq profiles and supervised hierarchical clustering analysis for OXA-S and OXA-R HCC patients. Significantly differentially expressed transcripts matching the threshold (more than 2- fold difference) and the statistical analysis standard adjusted P value < 0.05 were selected. (D) Volcano plot shows the differentially express genes identified from RNA-seq analysis of HCC patient samples treated with OXA-based HAIC (OXA-S VS OXA-R). (E) Volcano plot shows the differentially express genes identified from RNA-seq analysis of HCC patient samples (NASH HCC VS Non-NASH HCC). (F) Schematic representation of the generation of OXA-R cell line. (G) The relative viability curves of MHCC-97H cells in OXA-P and OXA-R cells treated with different concentrations of oxaliplatin for 48h. (H) Apoptosis analysis of the apoptotic cells in OXA-P and OXA-R MHCC-97H cells under the treatment of OXA (20 μM, 48h). [file 13046_2024_3134_MOESM2_ESM.pdf]

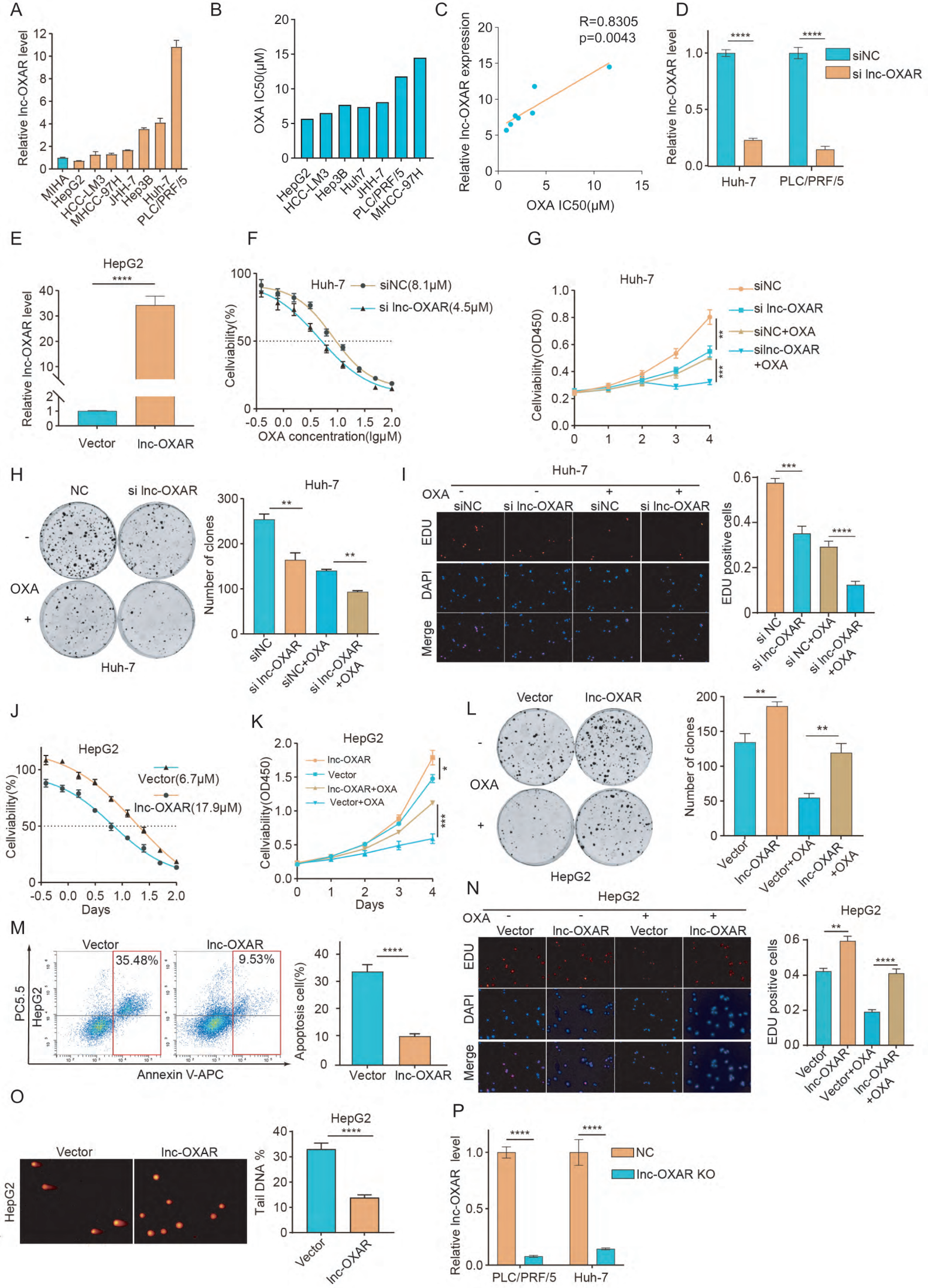

Supplement: Supplementary file 3 — Additional file 3: Supplementary Fig 2. lnc-OXAR promoted OXA resistance in NASH HCC. (A) The relative expression of lnc-OXAR in HCC cell lines and MIHA normal human hepatocyte cell line. lnc-OXAR was detected by qPCR and normalized to β-actin. (B) IC50 values of seven HCC cell lines. (C) Scatterplot analyzing the correlation between IC50 values and lnc-OXAR expression levels in the seven HCC cell lines. (D) lnc-OXAR expression in HCC cells transduced with si lnc-OXAR or NC. (E) lnc-OXAR expression in HCC cells transduced with vector or lnc-OXAR OE. (F) The relative viability curves of Huh-7 cells treated with different concentrations of oxaliplatin for 48 h after transfection with siNC and si lnc-OXAR. (G) Cell proliferation was assessed by CCK8 assays in Huh-7 cells transfected with siNC and si lnc-OXAR. (H) Colony formation assays and statistical analysis of Huh-7 cells transduced with siNC or si lnc-OXAR. (I) EdU detection in Huh-7 cells transfected with siNC and si lnc-OXAR with or without OXA treatment. Scale bar = 20μm. (J) The relative viability curves of HepG2 cells treated with different concentrations of oxaliplatin for 48 h after transfection with Vector and lnc-OXAR OE. (K) Cell proliferation was assessed by CCK8 assays in HepG2 cells transfected with Vector and lnc-OXAR OE. (L) Colony formation assays and statistical analysis of HepG2 cells transduced with Vector and lnc-OXAR OE. (M) The effect of lnc-OXAR overexpression on apoptosis in HepG2 cells treated with oxaliplatin (20μM, 48h). (N) EdU detection in HepG2 cells transfected with Vector and lnc-OXAR with or without OXA treatment. Scale bar = 20μm. (O) Representative images of comet assay of HepG2 cells transfected with vector and lnc-OXAR with OXA treatment (20μM, 48h) and quantitative analysis. Scale bar = 20 μm. (P) lnc-OXAR expression in HCC cells transduced with sg lnc-OXAR or NC. [file 13046_2024_3134_MOESM3_ESM.pdf]

A

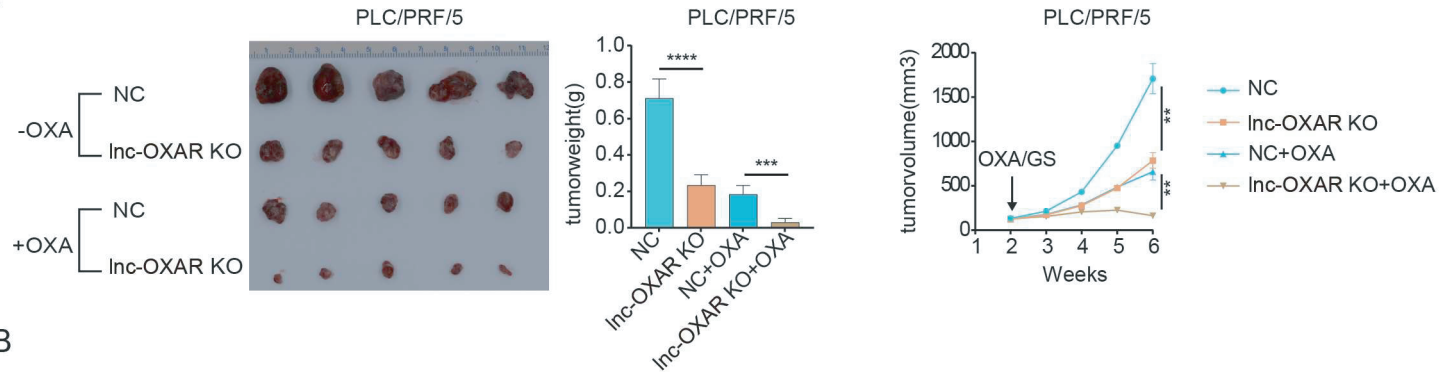

B

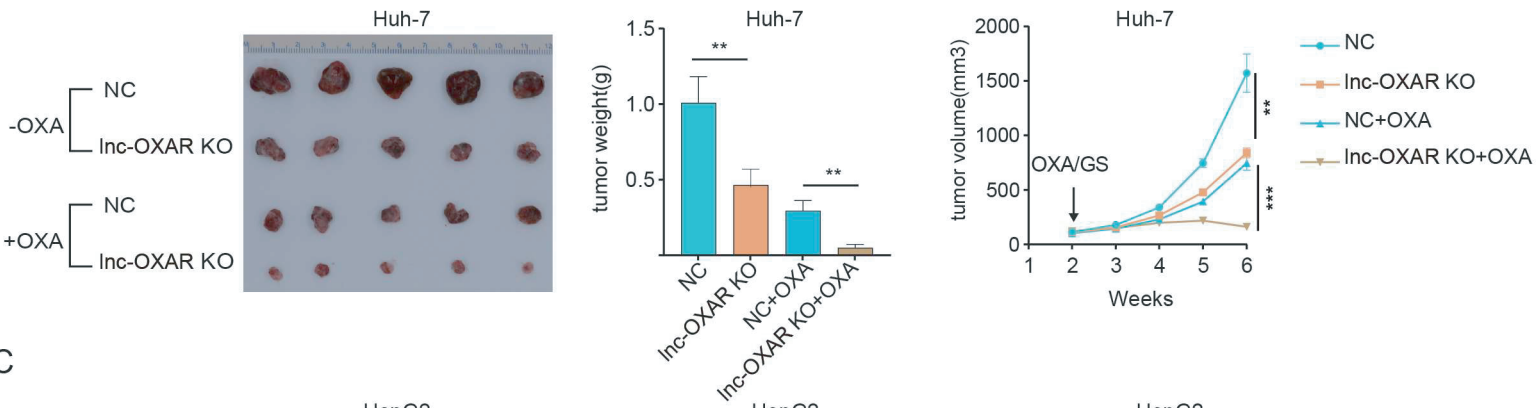

C

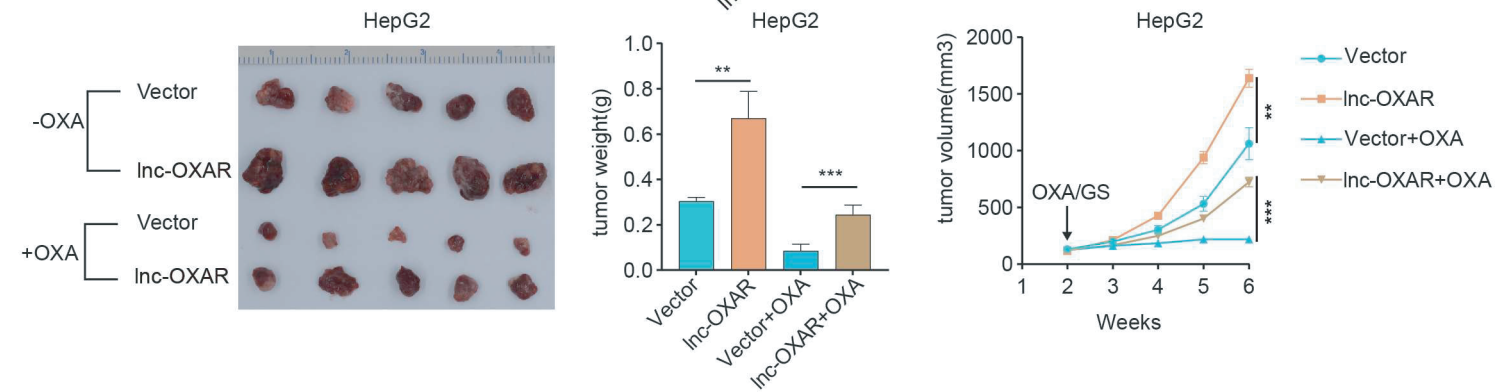

Supplement: Supplementary file 4 — Additional file 4: Supplementary Fig 3. lnc-OXAR promotes OXA resistance in vivo. (A) The effect of lnc-OXAR KO on the tumor growth of subcutaneously implanted PLC/PRF/5 cells treated with OXA (10 mg/kg) or vehicle control in nude mice (n=5). Scale bars, 1 cm. The tumor weights at the end points and measurement of tumor volumes weekly. GS, glucose saline. (B) The effect of lnc-OXAR KO on the tumor growth of subcutaneously implanted Huh-7 cells treated with OXA (10 mg/kg) or vehicle control in nude mice (n=5). Scale bars=1 cm. The tumor weights at the end points and measurement of tumor volumes weekly. (C) The effect of lnc-OXAR OE on the tumor growth of subcutaneously implanted Huh-7 cells treated with OXA (10 mg/kg) or vehicle control in nude mice (n=5). Scale bars=1 cm. The tumor weights at the end points and measurement of tumor volumes weekly. [file 13046_2024_3134_MOESM4_ESM.pdf]

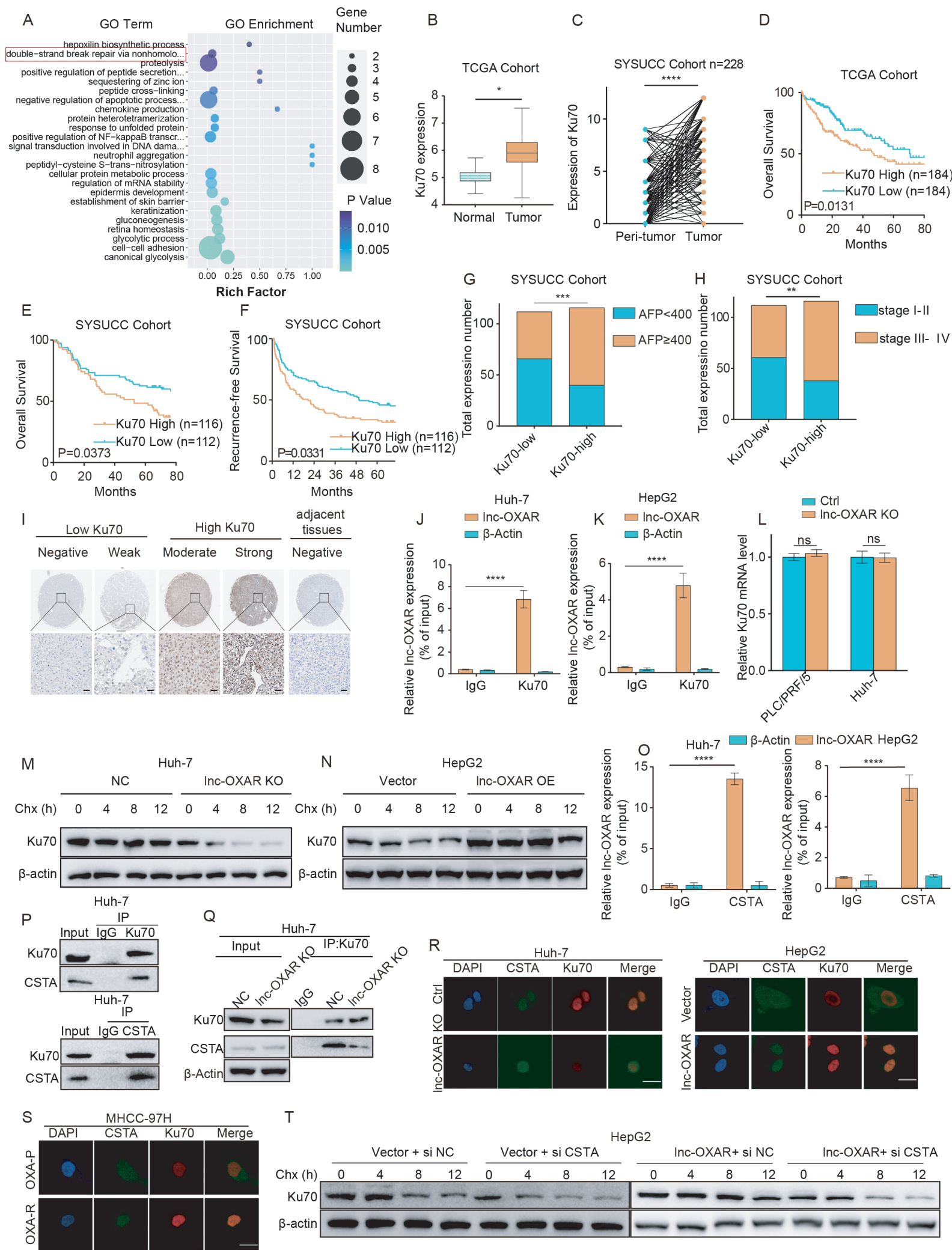

Supplement: Supplementary file 5 — Additional file 5: Supplementary Fig 4. lnc-OXAR maintained Ku70 stability by recruiting CSTA. (A) Enriched GO pathways of lnc-OXAR-binding proteins screened by ChIRP-MS assays (unique peptide ≥2, fold change >1.5). (B) The mRNA level of Ku70 between peritumor and tumor samples in TCGA cohort. (C) The IHC score of Ku70 in paired peritumor and tumor samples (n=228 pairs) in SYSUCC cohort. (D) Overall Survival rate of HCC patients categorized according to median Ku70 expression in TCGA cohort. (E) OS rate of HCC patients categorized according to median Ku70 expression in SYSUCC cohort. (F) RFS rate of HCC patients categorized according to median Ku70 expression in SYSUCC cohort. (G) The distribution of AFP (<400, ≥400) determined by the IHC score of Ku70 in the high or low groups in SYSUCC cohort. p values were determined by two-tailed Chi-square test. (H) The distribution of stage (I-II, III-IV) determined by the IHC score of Ku70 in the high or low groups in SYSUCC cohort. (I) Representative IHC staining images of Ku70 expression HCC tissues. Scale bar = 100 μm. (J) Ku70 RIP-qPCR analysis of lnc-OXAR level in Huh-7 cells. (K) Ku70 RIP-qPCR analysis of lnc-OXAR level in HepG2 cells. (L) Ku70 mRNA level in HCC cells after lnc-OXAR KO. (M) WB showing Ku70 protein in Huh-7 cells with or without lnc-OXAR KO treated with CHX for the indicated time. (N) WB showing Ku70 protein in HepG2 cells with or without lnc-OXAR OE treated with CHX for the indicated time. (O) CSTA RIP-qPCR analysis of lnc-OXAR level in Huh-7 and HepG2 cells. (P) WB results verified that Ku70 associated CSTA. (Q) WB of immunoprecipitated Ku70 and CSTA to determine the effect of lnc-OXAR8 KO on Huh-7 cells. (R) &(S) IF staining showed the co-localization of CSTA (green) and Ku70 (red) in HCC cells. Scale bar = 10 μm. (T) WB showing the effect of CSTA silencing on Ku70 protein in HepG2 cells with or without lnc-OXAR OE after treated with CHX for the indicated time. [file 13046_2024_3134_MOESM5_ESM.pdf]

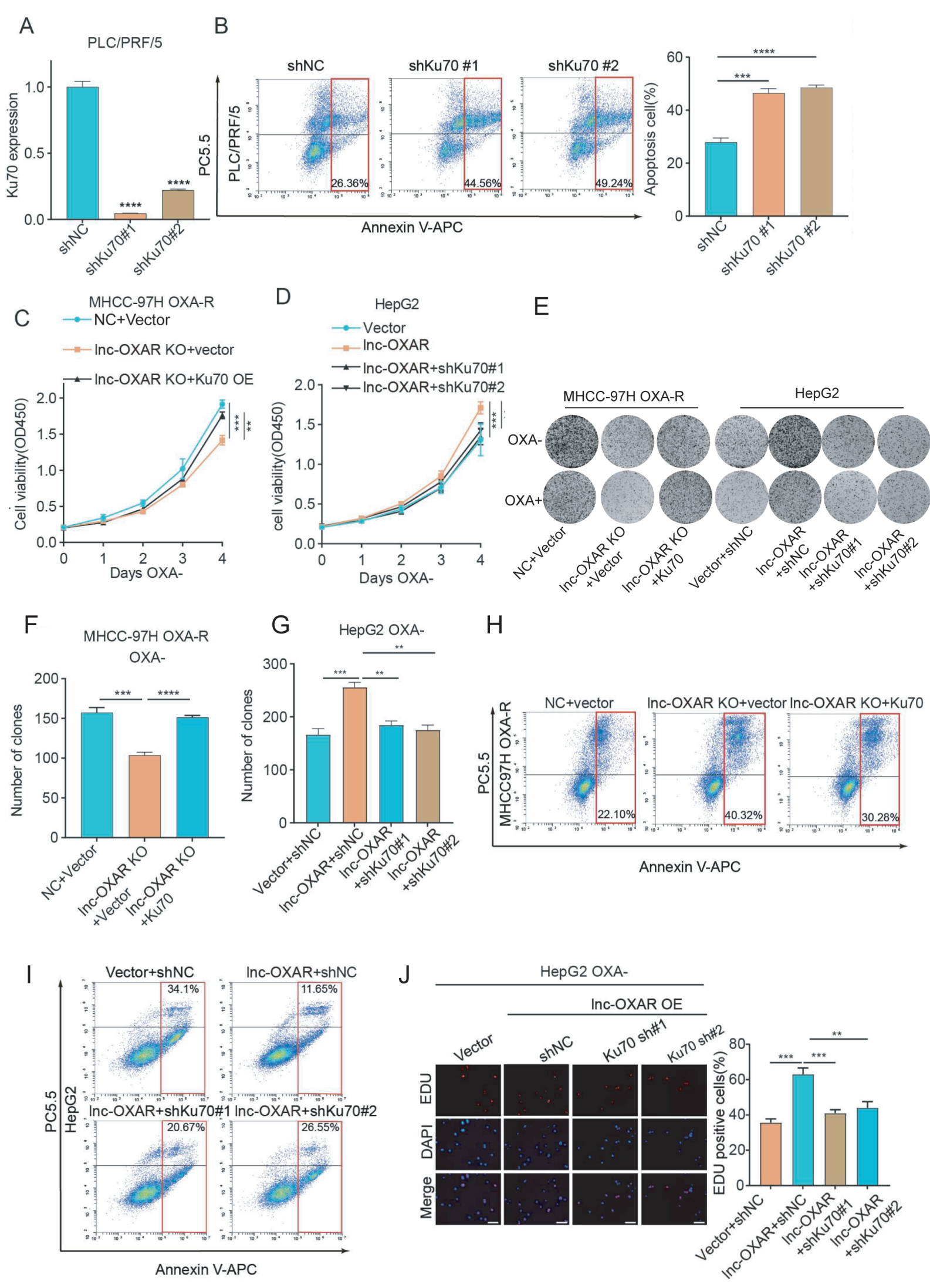

Supplement: Supplementary file 6 — Additional file 6: Supplementary Figure 5. The effect of lnc-OXAR on OXA resistance is dependent on Ku70. (A) Validation of Ku70 KD in PLC/PRF/5 cells. (B) The effect of Ku70 KD on the apoptosis of HCC cells with treatment (20 μM,48h) using flow cytometry analysis of Annexin V staining. (C) The effect of Ku70 OE on the CCK8 assay of lnc-OXAR KO in OXA-R cells without OXA treatment. (D) The effect of Ku70 KD on the CCK8 assay of lnc-OXAR OE in OXA-R cells without OXA treatment. (E) Images of colony formation assays in HCC cells with or without OXA treatment. (F) The effect of Ku70 OE on the apoptosis of lnc-OXAR-KO in OXA-R cells with treatment (20 μM,48h) using flow cytometry analysis of Annexin V staining. (G) The effect of Ku70 KD on the apoptosis of lnc-OXAR-overexpressing in HepG2 cells with treatment (20μM,48h) using flow cytometry analysis of Annexin V staining. (H) The effect of Ku70 OE on the colony-formation of lnc-OXAR KO in OXA-R cells with or without OXA treatment. (I) The effect of Ku70 KD on the colony-formation of lnc-OXAR-overexpressing in HepG2 cells with or without OXA treatment. (J) The effect of Ku70 KD on the EDU assay of lnc-OXAR-overexpressing in HepG2 cells without OXA treatment. Scale bar = 50 μm. [file 13046_2024_3134_MOESM6_ESM.pdf]

A

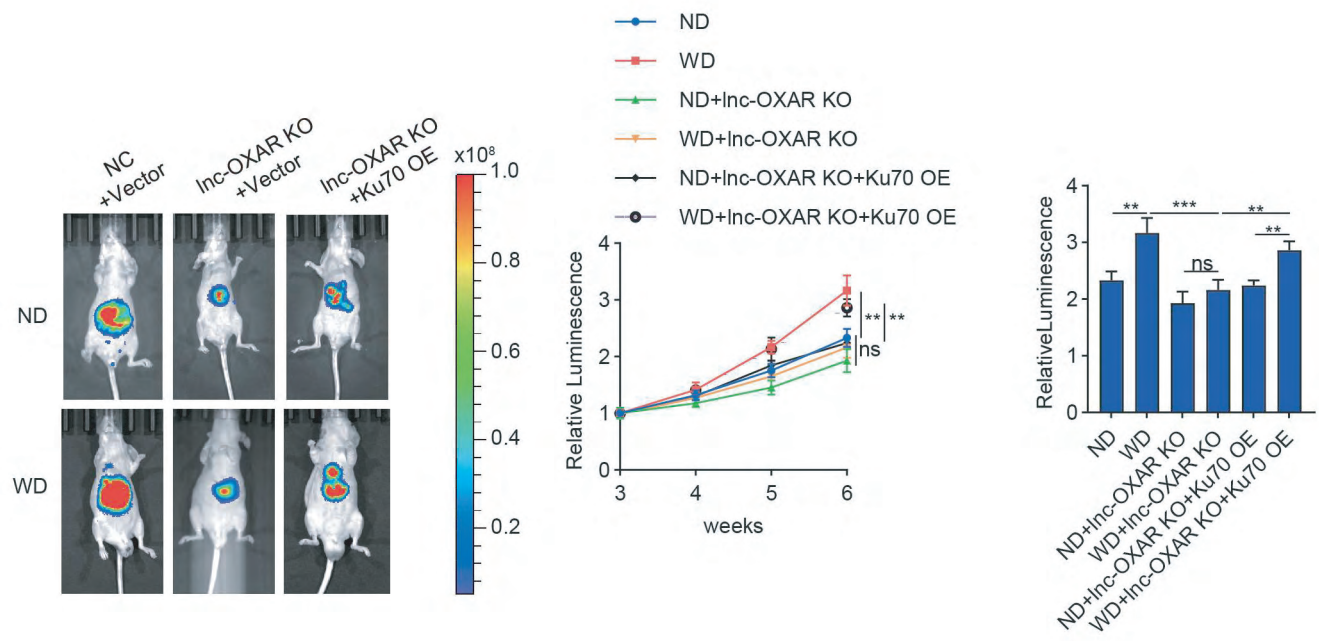

B

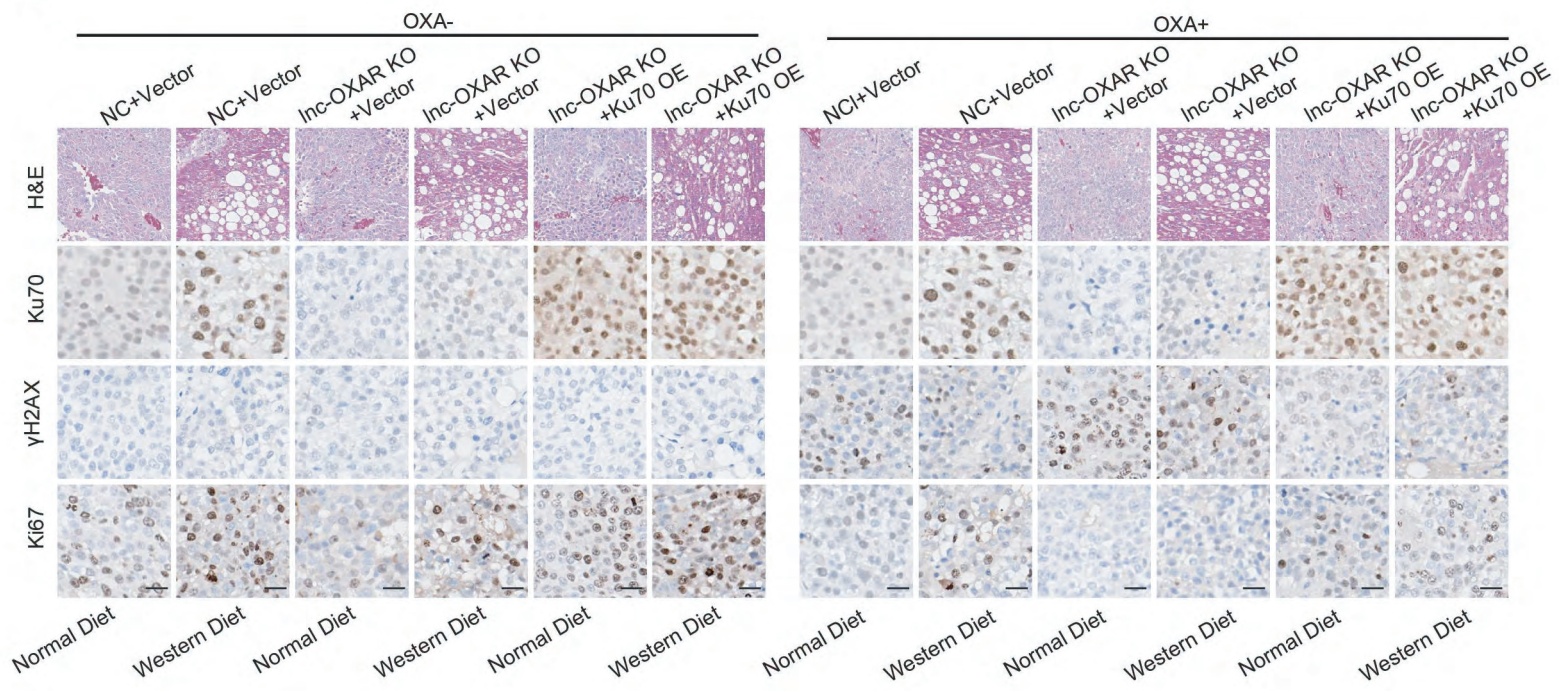

Supplement: Supplementary file 7 — Additional file 7: Supplementary Fig 6. The effect of lnc-OXAR on OXA resistance is dependent on Ku70. (A) Validation of Ku70 KD in PLC/PRF/5 cells. (B) HE staining and IHC staining of Ki67, Ku70 and γH2AX in the tumors. Representative images of four xenografts from each group are shown. Scale bar = 50 μm. [file 13046_2024_3134_MOESM7_ESM.pdf]

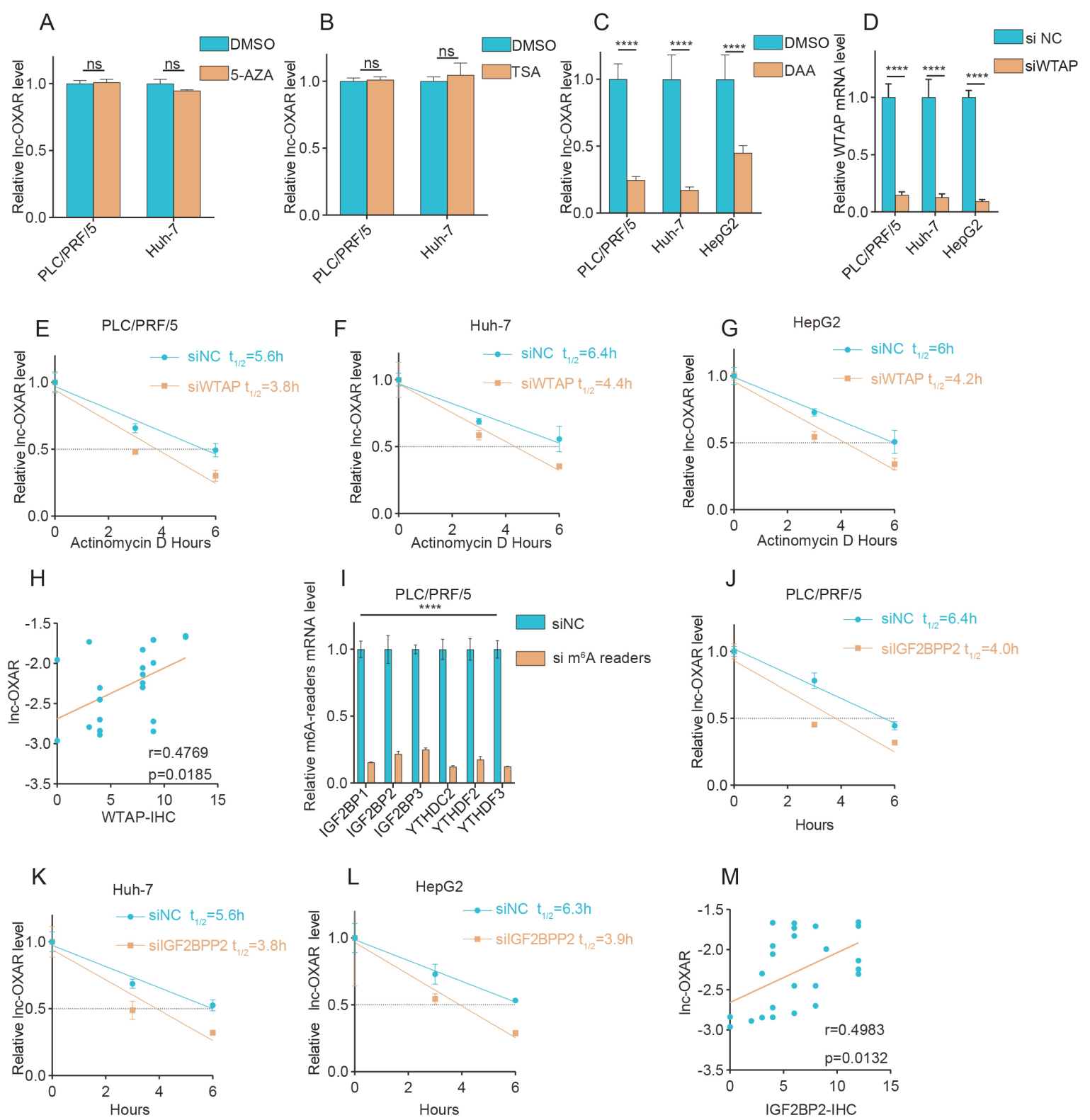

Supplement: Supplementary file 8 — Additional file 8: Supplementary Fig 7. m6A modification mediated lnc-OXAR upregulation in NASH-HCC. (A) lnc-OXAR expression level was detected after treated with 5-AZA in PLC/PRF/5 and Huh-7 cells. (B) lnc-OXAR expression level was detected after treated with TSA in PLC/PRF/5 and Huh-7 cells. (C) lnc-OXAR expression in HCC cells treated with 50 μM DAA or vehicle. (D) mRNA validation of WTAP knockdown in HCC cells. (E) The degradation rate of lnc-OXAR after WTAP silencing in PLC/PRF/5 cells with actinomycin D. (F) The degradation rate of lnc-OXAR after WTAP silencing in Huh-7 cells with actinomycin D. (G) The degradation rate of lnc-OXAR after WTAP silencing in HepG2 cells with actinomycin D. (H) Correlation between lnc-OXAR and WTAP expression in HCC patients from SYSUCC. (I) mRNA validation of six m6A readers’ knockdown in HCC cells. (J) The degradation rate of lnc-OXAR after IGF2BP2 silencing in PLC/PRF/5 cells with actinomycin D. (K) Degradation rate of lnc-OXAR after IGF2BP2 silencing in Huh-7 cells with actinomycin D. (L) Degradation rate of lnc-OXAR after IGF2BP2 silencing in HepG2 cells with actinomycin D. (M) Correlation between lnc-OXAR and IGF2BP2 expression in HCC patients from SYSUCC. [file 13046_2024_3134_MOESM8_ESM.pdf]
